# Using hydrologic landscape classification and climatic time series to assess hydrologic vulnerability of the western U.S. to climate

**DOI:** 10.5194/hess-25-3179-2021

**Published:** 2021-06-11

**Authors:** Chas E. Jones, Scott G. Leibowitz, Keith A. Sawicz, Randy L. Comeleo, Laurel E. Stratton, Philip E. Morefield, Christopher P. Weaver

**Affiliations:** 1 Oak Ridge Institute for Science and Education (ORISE), c/o U.S. Environmental Protection Agency, Center for Public Health and Environmental Assessment, Pacific Ecological Systems Division, 200 SW 35th St., Corvallis, OR 97333, USA; 2 U.S. Environmental Protection Agency, Center for Public Health and Environmental Assessment, Pacific Ecological Systems Division, 200 SW 35th St., Corvallis, OR 97333, USA; 3 c/o U.S. Environmental Protection Agency, Center for Public Health and Environmental Assessment, Pacific Ecological Systems Division, 200 SW 35th St., Corvallis, OR 97333, USA; 4 U.S. Environmental Protection Agency, Center for Public Health and Environmental Assessment, Health and Environmental Effects Assessment Division, Washington, DC 20460, USA; 5 U.S. Environmental Protection Agency, Center for Public Health and Environmental Assessment, Health and Environmental Effects Assessment Division, Research Triangle Park, NC 27709, USA; a currently at: Affiliated Tribes of Northwest Indians, Corvallis, OR 97333, USA; b currently at: AIR Worldwide, 131 Dartmouth Street #4, Boston, MA 02116, USA

## Abstract

We apply the hydrologic landscape (HL) concept to assess the hydrologic vulnerability of the western United States (U.S.) to projected climate conditions. Our goal is to understand the potential impacts of hydrologic vulnerability for stakeholder-defined interests across large geographic areas. The basic assumption of the HL approach is that catchments that share similar physical and climatic characteristics are expected to have similar hydrologic characteristics. We use the hydrologic landscape vulnerability approach (HLVA) to map the HLVA index (an assessment of climate vulnerability) by integrating hydrologic landscapes into a retrospective analysis of historical data to assess variability in future climate projections and hydrology, which includes temperature, precipitation, potential evapotranspiration, snow accumulation, climatic moisture, surplus water, and seasonality of water surplus. Projections that are beyond 2 standard deviations of the historical decadal average contribute to the HLVA index for each metric. Separating vulnerability into these seven separate metrics allows stakeholders and/or water resource managers to have a more specific understanding of the potential impacts of future conditions. We also apply this approach to examine case studies. The case studies (Mt. Hood, Willamette Valley, and Napa–Sonoma Valley) are important to the ski and wine industries and illustrate how our approach might be used by specific stakeholders. The resulting vulnerability maps show that temperature and potential evapotranspiration are consistently projected to have high vulnerability indices for the western U.S. Precipitation vulnerability is not as spatially uniform as temperature. The highest-elevation areas with snow are projected to experience significant changes in snow accumulation. The seasonality vulnerability map shows that specific mountainous areas in the west are most prone to changes in seasonality, whereas many transitional terrains are moderately susceptible. This paper illustrates how HL and the HLVA can help assess climatic and hydrologic vulnerability across large spatial scales. By combining the HL concept and HLVA, resource managers could consider future climate conditions in their decisions about managing important economic and conservation resources.

## 1 Introduction

A stable and predictable water supply is imperative for food security, ecosystem sustainability, economic stability, and even national security (National Intelligence Council, 2012) and is related to the threats of increased flooding, droughts, wildfire, and more extreme temperatures (Mancosu et al., 2015; Mekonnen and Hoekstra, 2016). The recognition of the potential socio-ecological threats of climate change to the water supply is a critically important topic, and the development of planning tools that identify vulnerabilities to these systems could help decision-makers assess the risks of environmental changes imposed by climate as well as other contemporary risks (e.g., population growth and habitat conversion) (Glick et al., 2011; Lawler et al., 2010). Climatic and hydrologic change will not impact stakeholders equally across sectors, and thus the specific concerns and adaptation strategies of different industries threatened by those risks will vary. The hydrologic landscape vulnerability assessment described herein provides a relatively simple approach for assessing hydrologic vulnerability based upon inferences of hydrologic behavior (using hydrologic landscapes) in response to climatic impacts. This approach can be applied across large geographic regions and can potentially benefit numerous sectors, including environmental, economic, and other ecosystem services.

Numerous studies have examined projected changes in climate and hydrology on regional and national scales that relate to this study in the western United States (U.S.). Climate-related risk to snow-dominated areas and ski areas was identified by Nolin and Daly (2006) in the Pacific Northwest (PNW, which includes Washington, Oregon, and Idaho), whereas observations and modeled simulations for snow water equivalents (SWEs) were found to be similar in the western U.S. (Mote et al., 2005). Barnett et al. (2005) found potential climate-driven water supply deficits in snow-dominated areas around the globe. McAfee (2013) examined projected changes in potential evapotranspiration (PET, calculated using numerous methods) and found regional analyses to be more inconsistent than studies across the conterminous U.S., which indicated sensitivities to the methods used. Hill et al. (2013, 2014) predicted thermal vulnerability of streams and river ecosystems to climate across the U.S., while Battin et al. (2007) found that salmon habitat in snow-dominated streams was more vulnerable than habitat in lowland streams. The relevant analyses of Nijssen et al. (2001) on hydrologic sensitivity of rivers globally found (1) ubiquitous warming, with the greatest warming in winter months at higher latitudes, (2) more precipitation with high variability, (3) early to mid-spring snowmelt causing increased spring streamflow peak in the coldest basins, decreased spring runoff, and increased winter runoff in transitional basins, and 4) increased annual streamflow with high-latitude basins. While snow-fed streams in the western U.S. seem less likely to change flow regimes, perennial and intermittent rain-fed streams are more likely to change in flow regime (Dhungel et al., 2016). In response to droughts of the recent past, Mann and Gleick (2015) highlight the strong correlation between very hot years and very dry years; thus as temperatures increase at the upper extreme, precipitation is becoming more scarce. A study by Cook et al. (2015) found a growing risk of unprecedented drought in the western U.S. based on temperature projections and no clear pattern in future precipitation. This sampling of the existing research highlights the cross-cutting hydrological changes that are occurring across the nation and illustrates how different sectors and geographies are experiencing different impacts.

“Vulnerability” has been defined in many ways, depending upon discipline and application (Adger, 2006; Füssel, 2007). Vulnerability assessments often integrate exposure, sensitivity, and adaptive capacity to stressors (Adger, 2006; Füssel, 2007; Füssel and Klein, 2006; IPCC, 2014). Researchers have studied vulnerability at varying scales across numerous regions for a diversity of stakeholders, and they tend to focus on the most relevant metrics for their particular application (Farley et al., 2011; Glick et al., 2011; IPCC, 2014; Nolin and Daly, 2006; U.S. Global Change Research Program, 2011; Watson et al., 2013). However, better products and services are needed to enable local communities to plan for and respond to hydrologic change, which includes services that improve understanding, observing, forecasting, and warning about significant hydrologic events (Tansel, 2013). Glick et al. (2011) and Lawler et al. (2010) both emphasize the importance to managers of understanding the potential impacts of climate on the resources that they manage.

There have been many efforts to assess hydrologic vulnerability related to specific stakeholders, ecosystems, or locations. For example, Vörösmarty et al. (2000) examined the vulnerability of global water resources to changes in climate and population growth. Hill et al. (2014) assessed stream temperature vulnerability to climate for sites across the U.S. In another example, Winter (2000) suggested that the vulnerability of wetlands to changes in climate depended upon their position within the hydrologic landscape.

There are opportunities to build upon previous efforts to map hydrologic vulnerability across large geographic areas while creating tools that stakeholders may use to understand the potential impacts for their asset of interest in specific watersheds. Winter (2001) described the concept of classifying the physical landscape and climatic properties of large landscape units based on hydrologic landscape (HL). Surface water and groundwater availability in watersheds is impacted by differences in geology, terrain, soils, seasonal temperature patterns, precipitation magnitude, and precipitation timing (Tague et al., 2013; Winter, 2001) and is not uniform across regions (Hamlet, 2011; Jung and Chang, 2012; Tague and Grant, 2004). Catchments that share similar key physical and climatic characteristics are expected to have similar hydrologic characteristics; i.e., surface water and groundwater interactions, deposition, timing, and accumulation of precipitation, surface runoff patterns, and groundwater flow (Nolin, 2011; Thompson and Wallace, 2001).

The HL concept has been applied to the U.S. using a clustering method (Wolock et al., 2004) to develop 20 non-contiguous regions, which were much larger than the catchment scale. Since that effort, modified approaches have not used clustering approaches but have used catchment-based classification in Oregon (Leibowitz et al., 2014; Patil et al., 2014; Wigington et al., 2013), Nevada (Maurer et al., 2004), the PNW (Comeleo et al., 2014; Leibowitz et al., 2016), and Bristol Bay, Alaska (Todd et al., 2017). In applying the HL approach in Oregon and the PNW, the clustering approach was abandoned for a conceptual approach based upon important factors known to contribute to hydrologic flow (Wigington et al., 2013), where two climatic factors and three landscape characteristics were categorized for each catchment; the resulting classification allows the estimation of catchment-scale hydrologic behavior across large spatial scales. The approach shows promise in predicting seasonal and monthly hydrologic patterns (Leibowitz et al., 2014). Leibowitz et al. (2014) adapted the classification system applied by Wigington et al. (2013) to illustrate the applicability of HLs at the watershed scale for representing normal (1971–2000) monthly average streamflow in three case study watersheds in Oregon. They used climate projections (2041–2070) to estimate hydrologic behavior of watersheds relative to 1971–2000. Leibowitz et al. (2016) expanded the approach and applied the HL classification to Oregon, Washington, and Idaho. The more recent studies using the hydrologic landscape classification approach have been applied at a watershed scale (Patil et al., 2014; Leibowitz et al., 2016; Todd et al., 2017).

A number of tactics have been used to investigate the influence of climate on hydrologic behavior (Luce and Holden, 2009; Safeeq et al., 2014; Vano et al., 2015). To extend the work previously completed from HL-based climate projections, we assess hydrologic vulnerability at the catchment scale by integrating the HL approach into an analysis of climatic variability. Our hydrologic landscape vulnerability approach (HLVA) provides spatially continuous, application-specific estimates of climatic vulnerability (maps of the HLVA indices). One of the benefits of the HLVA is to place recent and projected environmental changes in the context of available historic data. In the HLVA, we use proxies for the three components of vulnerability: (a) historic climate data and their derivatives as proxies for sensitivity (the sensitivity of a particular system to each variable); (b) climate projections as proxies for exposure (the future projected condition increases or decreases a system’s exposure to a change); and (c) qualitative considerations of ecosystems, stakeholders, or industries as proxies for adaptive capacity (the presence of a system in a location is indicative that the system has historically had sufficient adaptive capacity to exist in that area). Using HLVA, we examine vulnerability to changes in temperature, precipitation, potential evapotranspiration, snow accumulation, surplus water, climatic moisture, and seasonality of the water surplus. This method highlights areas that are projected to experience deviations from historic conditions to understand the patterns in magnitude, timing, and type of precipitation and the quantity and seasonality of available water at a catchment scale. These estimates of hydrologic vulnerability could offer important insight into the potential resilience of socially and economically valuable locations and stakeholders in an area.

We assess the hydrologic vulnerability of socially and economically valuable locations by applying the HL concept using climatic projections in the western U.S. We analyzed the output from the HL analyses to address three research objectives: (1) develop an index of vulnerability based on climate; (2) map areas that are projected to be more vulnerable to environmental change; and (3) determine the vulnerability indices for socially and economically valuable locations, including three example case studies for regional industries that are economically important in the region. By integrating the concept of hydrologic landscape classification, hydrologic vulnerability, and climatic impacts, this study lays the groundwork for making spatially explicit generalizations about the hydrologic vulnerability of socially and economically valuable locations across large landscapes.

## 2 Methods

### 2.1 Study area

**Figure 1.**
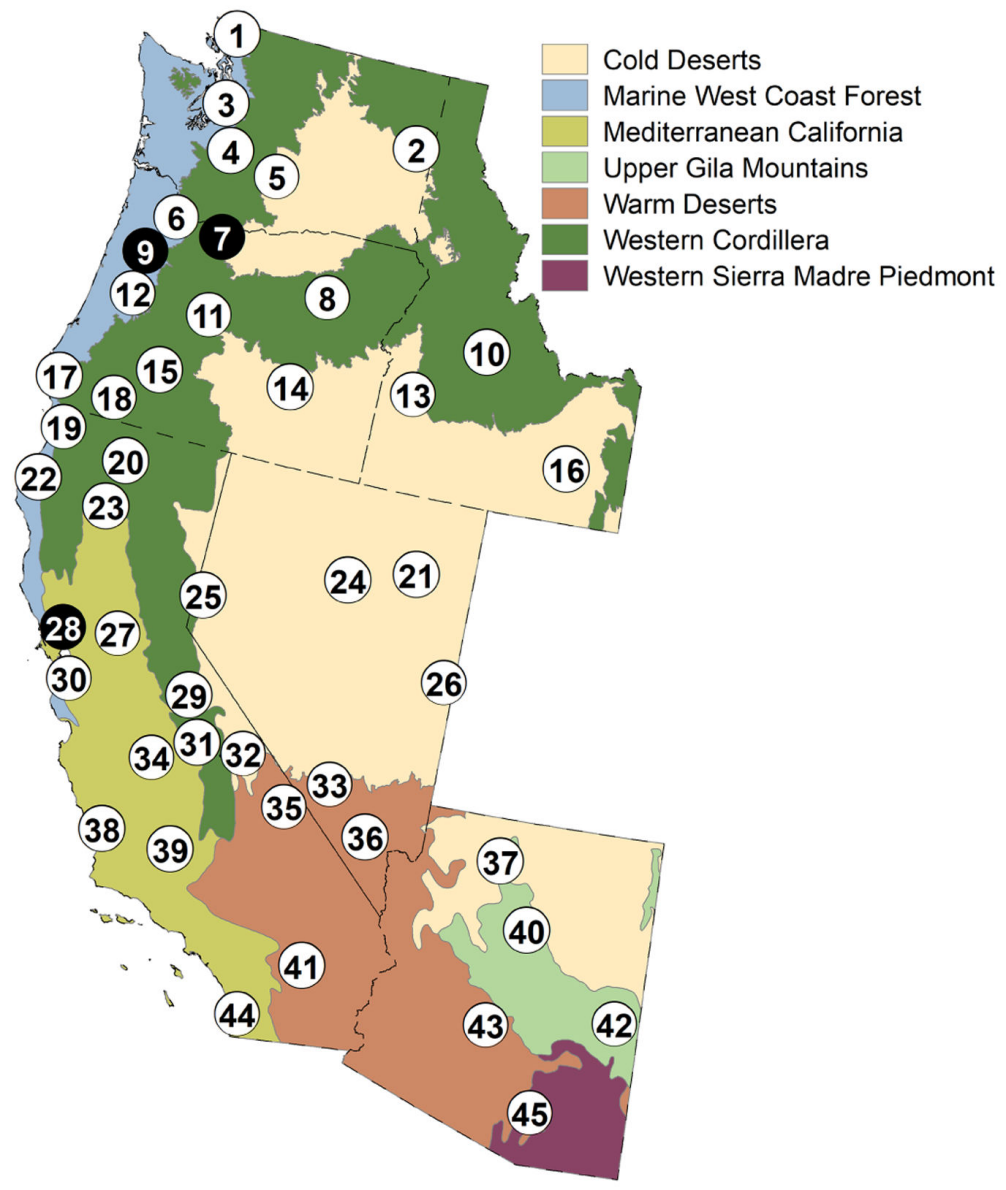
Study area showing a map with the six states of WA, OR, ID, CA, NV, and AZ. Also shown are the seven EPA Level II Ecoregions (https://www.epa.gov/eco-research/ecoregions-north-america, last access: 3 June 2021) and 45 locations identified by numbered circles with three case study locations in black circles (Table 2). State boundaries are indicated by black dashed lines.

The study area includes the states of Washington, Oregon, Idaho, California, Nevada, and Arizona in the western U.S. (Fig. 1). These states extend across a wide range of climates and diverse physiographic settings. The lowest elevation across the six states is 85m below sea level (Death Valley, California), while the highest elevation is 4421m above sea level (Mt. Whitney, California) (U.S.G.S. National Elevation Dataset available at https://nationalmap.gov/elevation.html last access: 3 June 2021). The Sierra Nevada mountain range is oriented in a north–south direction near the eastern border of California and transitions to the Cascade mountain range that is oriented north–south through Oregon and Washington (US Topo Quadrangles available at https://nationalmap.gov/ustopo). There are numerous other mountain ranges in the other states as well. The Sierra Nevada and Cascade mountain ranges generate orographic effects that cause upwind areas to the west to have greater precipitation relative to the downwind, eastern regions (Dettinger et al., 2004; Siler et al., 2013). High-elevation areas receive most of their precipitation as snow (Brekke et al., 2009; Mote et al., 2005), while lowland and coastal areas receive predominantly rain (Brekke et al., 2009; Mock, 1996), but much of the study area receives a balance of snow and rain. The topographic differences drive precipitation patterns across the area and cause differences in the total annual precipitation or the seasonality of maximum precipitation (Mock, 1996). In the arid southwest, summer monsoons deliver most of the annual precipitation, whereas in the PNW, winter rains and snows prevail (Mock, 1996). However, the western U.S. is regularly affected by atmospheric rivers that deliver large quantities of rain or snow over short periods (Dettinger, 2011; Hidalgo et al., 2009). The seasonal variability of surface air temperature varies widely across the study area. Portions of each state are classified as deserts with summer maximum temperatures regularly exceeding 40 °C (NOAA State Climate Extremes Committee, 2016). Each state has also recorded temperatures less than −40 °C (NOAA State Climate Extremes Committee, 2016). Some areas have mild climates with little seasonal variation in temperature (Daly, 2016b). Geology in the study area varies from high-permeability sedimentary deposits or relatively recent volcanic deposits to low-permeability igneous metamorphic and sedimentary formations and older volcanics (Comeleo et al., 2014; Stratton et al., 2016).

### 2.2 Hydrologic landscape classification

Assessment units (AUs) are aggregations of NHDPlusV2 catchments (McKay et al., 2012) that were grouped to have a target area of 80 km^2^, as described in Leibowitz et al. (2016). In this study, the same assessment units used in the Leibowitz et al. (2016) study have been used and their method applied to the expanded six-state study region to delineate 29 097 assessment units for the study’s expanded six-state study region. For this analysis, we retain an AU if its centroid was located within the boundary of our project area or if the AU extended across an international boundary. All AU polygons are clipped to the international boundary of the U.S. These conditions allow us to avoid edge effects at international and state borders by avoiding overlapping AUs at state boundaries and analyzing the HLs up to all international borders.

**Figure 2.**
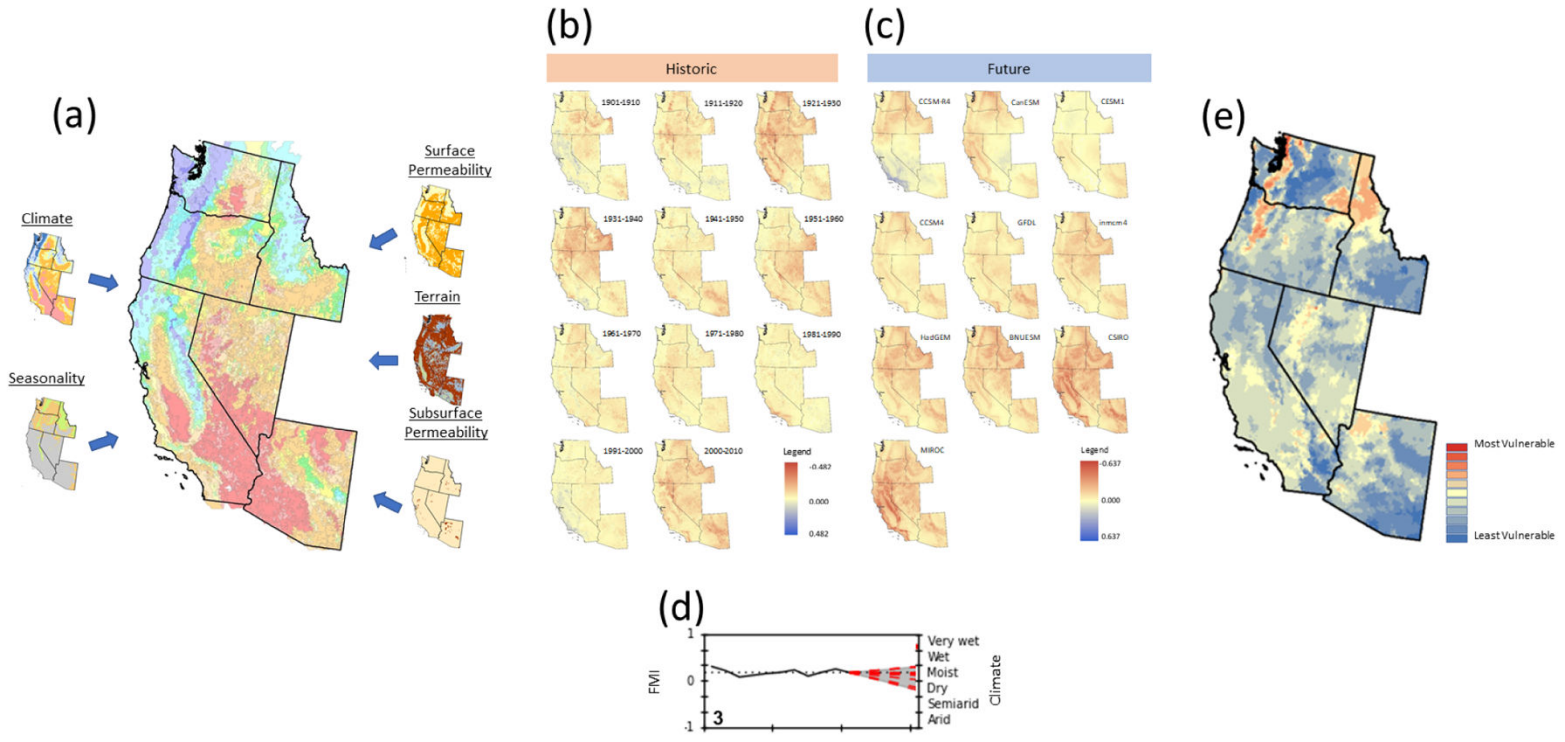
Mapping of hydrologic vulnerability. **(a)** A hydrologic landscape map is developed for six western states using 1971–2000 normals for climate (Feddema Moisture Index; FMI) and seasonality, along with surface permeability, terrain, and subsurface permeability geophysical data. **(b)** Historical decadal analysis is run from 1901 through 2010 for each of seven metrics: monthly temperature, precipitation, potential evapotranspiration, surplus water, snow water equivalent, FMI (shown), and seasonality. **(c)** Future predicted behavior is estimated for each of the seven metrics, based on 10 climate model projections (FMI shown). **(d)** Vulnerability is then defined as the number of climate projections that lie outside of the historical 2 standard deviation threshold (example for FMI from Napa–Sonoma shown). **(e)** Vulnerability values are then mapped for each metric across the six-state study area (FMI shown).

Building upon Winter’s (2001) approach and the Wolock et al. (2004) clustering approach, Wigington et al. (2013) developed their simple conceptual HL classification based on climatic and physical characteristics of the physical watershed. They combined five indices related to hydrologic flow (Fig. 2a) to characterize the major drivers that control the magnitude and timing of water movement through the landscape and into the groundwater or stream network: (1) climate, which describes the overall water availability, (2) seasonality of water surplus, which is the season when the maximum excess of water is available to infiltrate into the soil or flow as surficial runoff, (3) subsurface permeability, (4) terrain, and (5) surface permeability. Note that Wigington et al. (2013) referred to subsurface and surface permeability as aquifer and soil permeability, respectively. The five HL indices, described in more detail below (Sect. 2.2.1 through 2.2.5), are concatenated into a five-character HL code (e.g., WsLMH, SwHTH, or DfHfL) that characterizes an AU.

Leibowitz et al. (2016) modified the Wigington et al. (2013) approach by including the use of assessment units based on National Hydrography Dataset Plus V2 catchments, a modified snowmelt model that was validated over a broader area, a subsurface permeability index that does not require pre-existing aquifer permeability maps, and a surface permeability threshold based on objective criteria. Using this modified method (herein described as the modified Wigington et al., 2013, approach), they developed an HL map of the PNW. Here, we used the modified Wigington et al. (2013) approach to develop an HL classification of California, Nevada, and Arizona. This was then combined with the PNW map (Leibowitz et al., 2016) to create an HL map of the study area.

#### 2.2.1 Climate

The Wigington et al. (2013) approach derived the climate index from the Feddema Moisture Index (FMI) (Feddema, 2005):

(1) FMI={1−PETPifP≥PET,PPET−1ifP<PET,}

where FMI (Eq. 1) values range from −1.0 (arid) to 1.0 (very wet). *P* is the mean precipitation (mm) over a 30-year period, which is derived from climate data described in Sect. 2.3, and PET is the potential evapotranspiration (mm) calculated using the Hamon (1963) method that utilizes mean daily temperature, daytime length (calculated based on latitude), and a calibration coefficient. The range of FMI values was the basis for defining a climate index consisting of six classes: arid (A; −1.0 ≤ FMI < −0.66), semi-arid (S; −0.66 ≤ FMI < −0.33), dry (D; −0.33 ≤ FMI <0.0), moist (M; 0.0 ≤ FMI <0.33), wet (W; 0.33 ≤ FMI <0.66), and very wet (V; 0.66 ≤ FMI <1.0) (Wigington et al., 2013). FMI was calculated using regional precipitation and temperature rasters (described in Sect. 2.3) for each period of interest. The FMI value was then averaged over each AU.

#### 2.2.2 Seasonality

We used the Leibowitz et al. (2016) approach to develop a seasonality index that identifies the season of the maximum monthly average snowpack-corrected surplus water ($S_{m}^{\prime }$):

(2) $$S_{m}^{\prime }=S_{m}-\Delta {\text{PACK}}_{m}^{*},\;\;S_{m}^{\prime }=(P_{m}-{\text{PET}}_{m})-({\text{PACK}}_{m}^{*}-{\text{PACK}}_{m-1}^{*}),$$

where $S_{m}^{\prime }$ (Eq. 2) is the average snowpack-corrected water surplus (mm) for month m, *S*_m_ is monthly water surplus (*P* − PET), and *P*_m_ and PET_m_ are monthly precipitation and monthly PET, respectively. ${\text{PACK}}_{m}^{*}$ is a monthly bias-corrected snowpack value (in millimeters of SWE) restricted to values greater than zero, based on the Leibowitz et al. (2016) modifications to the Leibowitz et al. (2012) snowpack model. Note that $\Delta {\text{PACK}}_{m}^{*}$ can have negative values, which represents snowmelt. For each month, $S_{m}^{\prime }$ was calculated for the regional raster before identifying the month of maximum $S_{m}^{\prime }$ for the majority of pixels in each AU. The month of maximum $S_{m}^{\prime }$ was used to identify the season of maximum $S_{m}^{\prime }$ based upon four seasonality classes: fall (f; October–December), winter (w; January–March), spring (s; April–June), and summer (u; July–September). The PNW analysis by Leibowitz et al. (2016) only included two seasonality classes: summer seasonality did not occur, while fall and winter were combined into a winter class, since this represented the PNW’s wet season. For this analysis, winter and fall were separated and all four seasonality classes were used, because fall and winter are distinct seasons in other parts of the nation.

#### 2.2.3 Subsurface permeability

Leibowitz et al. (2016) utilized the Comeleo (2014) aquifer permeability dataset. We applied a similar approach to the Stratton et al. (2016) aquifer permeability datasets, which is herein referred to as subsurface permeability. Each dataset classifies the subsurface permeability into high- (H) and low-permeability (L) classes, which are assigned with a threshold of 8.5×10^−2^ md^−1^ hydraulic conductivity. Using these data, we analyzed the subsurface permeability of each AU by identifying the subsurface permeability class for the majority of pixels within each AU in California, Nevada, and Arizona.

#### 2.2.4 Terrain

To classify terrain, we used the same approach as Wigington et al. (2013). We analyzed a 30m digital elevation model to classify the landscape based upon the topographic characteristics of each AU. “Mountainous” (M) areas had AUs with <10% of the area identified as flat (<1% slope) and greater than 300m of total relief. AUs with more than 50% area with <1% slope were classified as “flat” (F). All other AUs were identified as “transitional” (T).

#### 2.2.5 Surface permeability

For surface permeability, the Leibowitz et al. (2016) HL approach utilized the STATSGO soil permeability raster developed by the Pennsylvania State University Center for Environmental Informatics (http://www.cei.psu.edu/, last access: 3 June 2021) for the top 10 cm of soil (Miller and White, 1998) in the conterminous U.S. The STATSGO soils database was selected because of its complete coverage of the conterminous U.S., despite SSURGO’s higher spatial resolution yet incomplete coverage of the study area. Leibowitz et al. (2016) identified whether the majority of each AU had high (H; >1.52 cm h^−1^) or low (L; ≤ 1.52 cm h^−1^) soil permeability. We applied the same approach to classify the surface permeability of each AU into two classes throughout the region.

### 2.3 Climate analyses

#### 2.3.1 Climate normal (1971–2000)

The climate normal was defined as the 1971–2000 period to align with the Leibowitz et al. (2016) study. Average monthly precipitation and mean temperature were acquired from Parameter-elevation Regressions on Independent Slopes Model (PRISM; Daly, 2016b) data for our normal climatic period at a resolution of approximately 400 m. The PRISM Climate Mapping Program is an ongoing effort to produce detailed, spatial climate datasets (Daly, 2016a; Daly et al., 2000). PRISM uses point measurements of climate data and a digital elevation model to map climate across the U.S. from 1895 to the present, including regions impacted by high mountains, rain shadows, temperature inversions, coastal regions, and associated complex meso-scale climate processes. Using ArcGIS (ESRI, 2016), the data were clipped to the project boundary and used to calculate the average for seven metrics: monthly temperature (°C), precipitation (mm), PET (mm), surplus water (mm), snow water equivalent (mm), the FMI climate index (unitless), and seasonality of water surplus (unitless). Each metric is an input to or products of the HL classification process.

#### 2.3.2 Historical climate analyses (1901–2010)

Unlike the 1971–2000 monthly precipitation and temperature data, a time series of gridded monthly historical climate data at a spatial resolution of 400m was not available without paying a fee. However, daily PRISM data were freely available at 4 km resolution, so we used these to develop the historical climate analyses for the 1901–2010 period. These gridded data for daily mean temperature and precipitation were clipped to the project boundary and averaged for each month over each decade (i.e., 1901–1910, 1911–1920, etc.). The data were then statistically downscaled to 400m using the delta method (Hijmans et al., 2005; Ramirez-Villegas and Jarvis, 2010) to match the spatial and temporal resolution of the climate normal data (using the 400m resolution, monthly PRISM climate normal for the 1971–2000 period as the high-resolution dataset). We acknowledge the inaccuracies and uncertainty imposed in the temperature and precipitation datasets by applying the downscaling functions to the original climate projections. While the 400m data clearly have greater resolution and less error than the 4 km data, these data were to be aggregated to assessment units with a mean area of 56 km^2^. In practice, the larger 4 km resolution of the downscaled historical analysis should still be appropriate for the scale of the assessment units, and thus the trade-offs were deemed acceptable and preferable for characterizing the hydrology and climate for these analyses with no additional budget requirements.

Based on the approaches described, the downscaled data were used to calculate the average monthly PET, surplus water, snow water equivalent, FMI, and seasonality of water surplus for each decade (Fig. 2b). Summary figures were generated from these data, depicting spatial distribution of climate and seasonality for each decade across the project area. These data were compared to the climate normals using spatially continuous time series analyses (Fig. S1).

#### 2.3.3 Future climate analyses (2041–2070)

**Figure 3.**
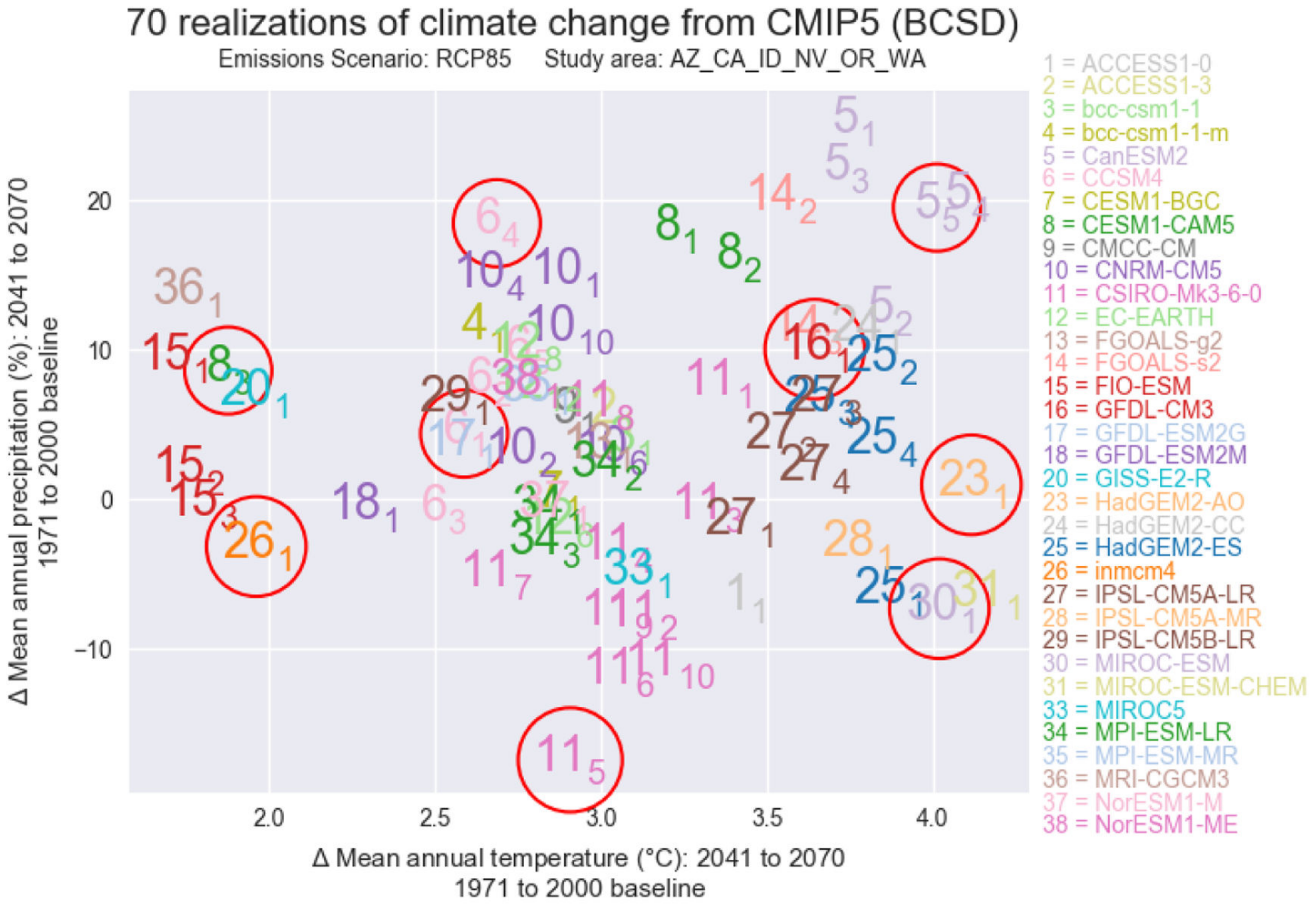
Scatterplot showing the range of mean temperature and precipitation projections for the 2041–2070 climate models across the study area. The circled data points identify the climate projections used in our analyses. Climate models are enumerated using the key to the right of the scatterplot. Subscripts denote the realization number of each unique projection. Legend colors are used to improve legibility where scatterplot symbols overlap.

In order to explore the potential range of modeled climatic response for the study area, we selected 10 climate model projections from the full ensemble of the World Climate Research Programme’s Coupled Model Intercomparison Project phase 5 multi-model ensemble climate dataset projections (WCRP CMIP5; https://esgf-node.llnl.gov/projects/esgf-llnl/, last access: 3 June 2021; Taylor et al., 2012). These models are based on the Representative Concentration Pathway (RCP) 8.5 emissions scenario, which assumes the highest rate of emissions into the 21st century and most closely relates to conditions observed to date (Schwalm et al., 2020). To reduce the complexity of the analyses, we used only this one emissions scenario. To select the specific model simulations to use in this study, we used the U.S. Environmental Protection Agency’s (EPA) LASSO tool (https://lasso.epa.gov/, last access: 3 June 2021; U.S. EPA, 2020) to generate a scatterplot comparing future temperature and precipitation change for the different CMIP5 models over the project area. Using the scatterplot and the approach described by the U.S. EPA (2020), we subjectively selected 10 models that spanned the entire range of predicted climatic responses of the full ensemble in a distributed manner (Fig. 3), including drier, wetter, colder, and warmer responses. Average monthly precipitation and temperature for the 10 projections (Table 1) were acquired from the monthly Bias Correction and Spatial Disaggregation (BCSD) archive (Bureau of Reclamation, 2014) for the 2041–2070 period. These data were clipped to the project boundary and resampled to a 400m grid using a bilinear approach (ESRI ArcGIS v10.4) to match the resolution and spatial extent of the climate data. The average monthly PET, surplus water, snow water equivalent, FMI, and seasonality of water surplus were calculated from the future climate data for each assessment unit. Example figures were generated that illustrate the spatial distribution of the differences in FMI (Figs. S1 and S2) and seasonality of water surplus (Figs. S3 and S4) from the normal period for each climate projection (Fig. 2c).

### 2.4 Mapping vulnerability indices

As discussed in the introduction, vulnerability can be measured by assessing the *exposure, sensitivity,* and *adaptive capacity* of a system to change (Adger, 2006; Füssel, 2007; Füssel and Klein, 2006; IPCC, 2014). Hydrology and climate are primary forcing factors for ecosystems (Nelson, 2005) and are critical to certain industries and stakeholders in particular areas, and thus analyses of historic variation in hydrology and climate in an area can serve as proxies for the historical *sensitivity* of those systems to environmental change. Likewise, we used future climate projections as a proxy for *exposure.* Projections that fell outside of historic observations were assumed to be associated with increased exposure to the forcing factors for environmental change, which include hydrology and climate. In terms of *adaptive capacity,* we assumed that the systems present in a location are adapted to the historic variability in conditions. We also assumed that the systems would become stressed by conditions far outside of those previously experienced. Further, we suggest that the greater the number of future climate projections that exceed or fall far below the historic range, the more vulnerable a system will be with respect to climate-induced changes. Thus, HLVA places projected environmental changes in the context of historic trends. The HLVA assesses vulnerability to changes in temperature, precipitation, potential evapotranspiration, surplus water, snow accumulation, climatic moisture, and seasonality of the water surplus by identifying areas that are projected to experience future deviations from historic conditions (Fig. 2e).

The 10 future climate projections (for the 2041–2070 period) were compared to the decadal averaged data from 1901 to 2010 for each AU. We calculated the historical standard deviation of each metric for each AU within the project area. For each metric, we assume that any projection within 2 standard deviations of the historical climate values does not contribute to an increase in vulnerability, whereas projections outside of that range increase the vulnerability. We then define vulnerability for a given metric as the number of the 10 projections that are outside of the historical 2 standard deviation threshold. Thus, the HLVA index assesses the likelihood that a given metric will exceed a 2 standard deviation threshold from the decadal mean under future climate scenarios. Because individual models exceed the threshold of 2 standard deviations from the mean in both the higher and lower directions, there is no unique direction of change associated with the vulnerability index. Thus, the vulnerability index, as defined, does not convey information about the projected direction of change. A vulnerability index of 10 indicates that all 10 climate projections were beyond 2 standard deviations from the historical mean and that the area is expected to experience projected conditions that it is not adapted to. The least vulnerable areas will have an index of zero, which indicates that all future climate projections fell within the 2 standard deviation threshold to which systems are adapted. The use of standard deviations is not an appropriate threshold metric for seasonality, because it is a categorical variable. For the seasonality metric, any projected seasonality value that has not been observed decadally between 1900 and 2010 increases the seasonality vulnerability index. For example, consider an AU that had predominantly experienced spring seasonality, with the occasional fall seasonality, and that 7 of 10 climate models project fall seasonality and 3 of 10 models predict winter seasonality for 2041–2070. Since winter seasonality was not observed for any decade between 1900 and 2010, the three predictions for winter seasonality would contribute to a vulnerability index of 3 for seasonality in that case. Finally, we analyzed the dominant HL code by area of the most vulnerable AUs (those having a vulnerability index greater than 7 on a scale of 10) for each metric in order to gain insight into the dominant HL characteristics that relate to hydrologic vulnerability.

### 2.5 Locational time series analyses

Forty-five locations (Fig. 1 and Table 2) were selected for potential applications of the HL approach to demonstrate the method’s relevance to potential water resource stakeholders to identify areas where we thought results could be of use to land managers. Specific sites were selected subjectively so that we could examine representative climate impacts at sites that may be of general interest. These sites include cities, national parks, mountains, national forests, and areas with hydrologically sensitive economic interests. AUs were used to represent a geographic feature if its centroid was located within the geographic boundary of a location of interest. The location boundary was defined by merging these AUs into a single polygon. For instance, the Great Basin National Park (GBNP) was covered by a single AU rather than numerous AUs because the centroid of only one AU was within the park boundary, whereas all other AU centroids were located outside of the GBNP boundary. The time series for the decadal averages for each of the climate-related HL metrics were analyzed for the AUs associated with each location. Decadal averages were plotted at the decadal midpoint for each 10-year period from 1901 to 2010. In addition, the 1971–2000 normal average for each variable and 10 climate projections (2041–2070) were also plotted. The HLVA was then used to determine the mean vulnerability index and the dominant HL code for the AUs associated with each location (Fig. 2d).

## 3 Results

### 3.1 Hydrologic landscape summary

**Figure A1.**
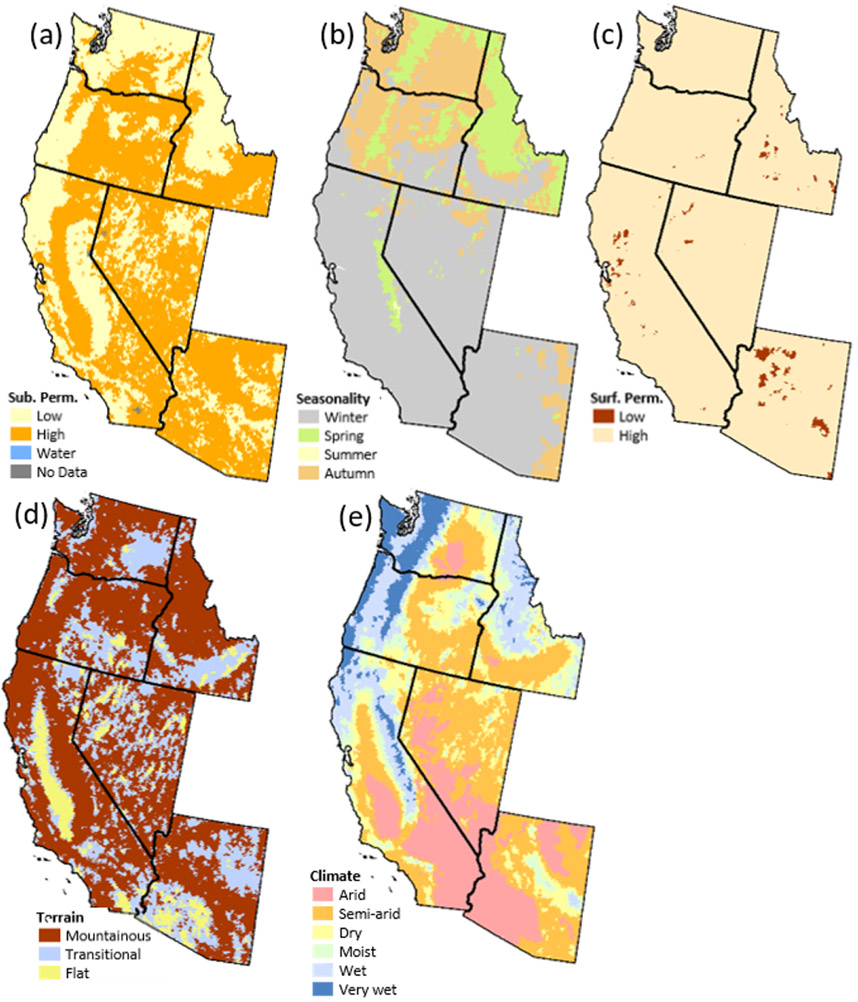
Component hydrologic landscape maps of Washington, Idaho, Oregon, California, Nevada, and Arizona were used in the analysis of the HLVA indices: **(a)** subsurface permeability, **(b)** seasonality of precipitation surplus, **(c)** surface permeability, **(d)** climate, and **(e)** terrain. Notes: the seasonality map for the PNW has been updated from the original Leibowitz et al. (2016) HL map, as we separated their winter seasonality into two seasons (winter and fall).

Table 3 shows the percent coverage of the HL categories for the six states. Thirty percent of the region is mountainous (elevation relief of AU >300m and <10% of AU area has slope <1%) and 7% is flat (AUs with more than 50% area having <1% slope). The remaining area is classified as transitional. According to the soil permeability dataset (Miller and White, 1998) produced from the STATSGO soils database (Soil Survey Staff, 2016), 98% of the surface soils (defined as the top 10 cm) are highly permeable (>4.23 μm s^−1^). Stratton et al. (2016) and Comeleo et al. (2014) classified the subsurface permeability of the six-state region as 60% high permeability and 40% low permeability. During the 1971–2000 climate normal period, most of the area has the highest monthly water availability (seasonality) during the winter (63%), followed by 24% of the area showing fall seasonality, 13% having spring seasonality, and only 1% experiencing summer seasonality. In addition, 30% of the area is classified as having a moist, wet, or very wet climate, while 70% is dry, semi-arid, or arid. The HL maps for the study area are included in the Appendix (Fig. A1). HL maps for the remainder of the conterminous U.S. are also available and are included in the Supplement (Fig. S6; although subsurface permeability maps are not available for all of the lower 48 states).

### 3.2 Climate vulnerability analyses

**Figure 4.**
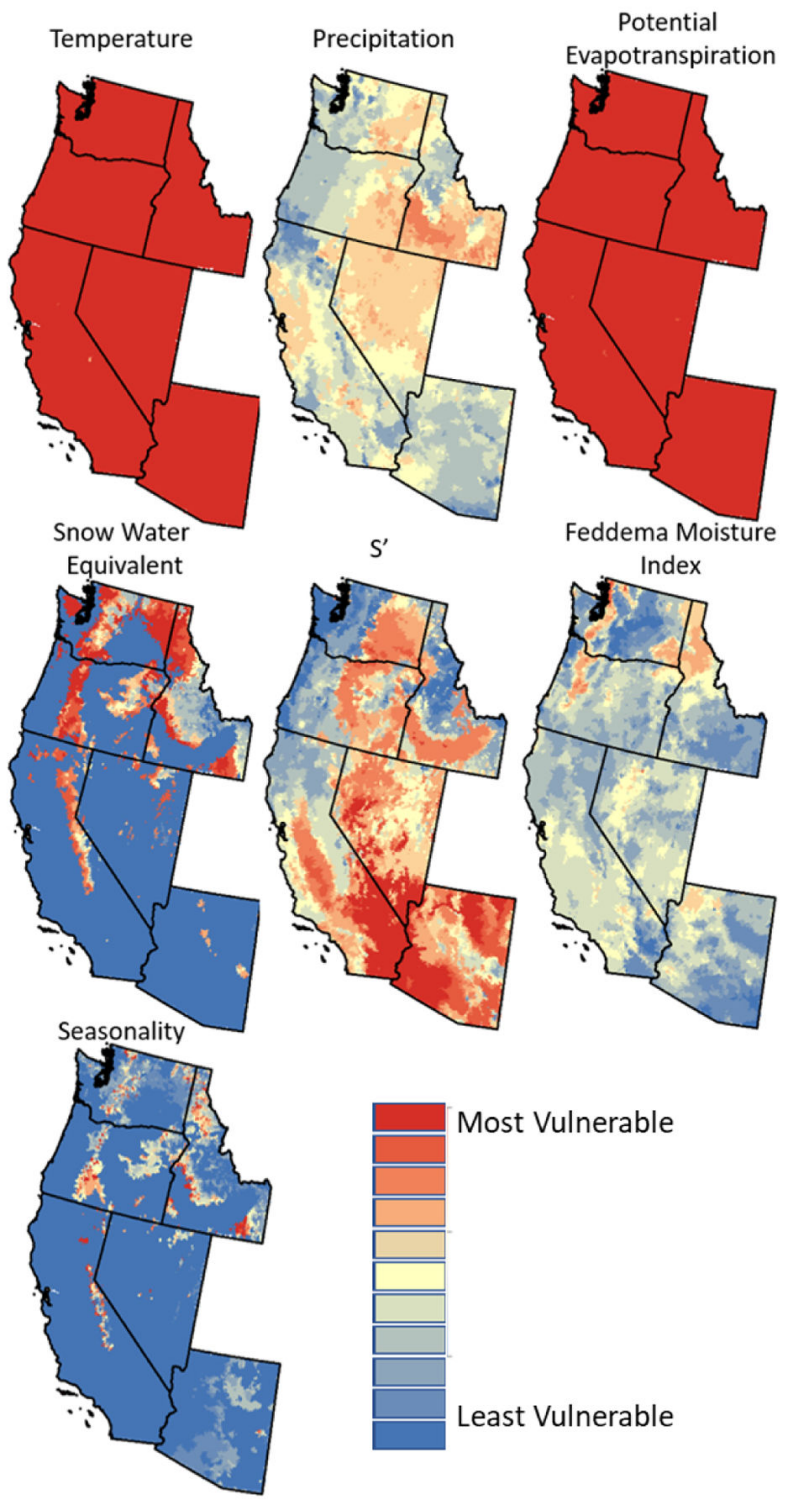
Vulnerability indices for temperature, precipitation, potential evapotranspiration, snow water equivalent (1 April), *S′* (available water), Feddema Moisture Index, and seasonality. The least vulnerable locations are those projected to be within 2 standard deviations of the historic (1901–2010) mean in all 10 climate models.

Using the analyses of historic and future climate, the vulnerability indices were mapped for all seven metrics (examples are provided for FMI and seasonality in the Supplement). The vulnerability maps (Fig. 4) identify areas that are subject to extreme future climatic and hydrologic variability (similar vulnerability maps for the conterminous U.S. are included in the Supplement, Fig. S6). Note that while it is possible to evaluate direction of change (greater than or less than 2 standard deviations) for the projection of an individual climate model, the vulnerability index is the integration of 10 individual models. Therefore, it is possible for individual models to exceed the threshold of 2 standard deviations from the mean in either the higher or lower directions; thus there is no unique direction of change associated with our vulnerability index as it has been defined.

All climate projections indicate that temperature will change almost ubiquitously across the Pacific west, indicating uniformly high vulnerability. However, changes in precipitation are much more spatially variable. The cold deserts and Mediterranean California Ecoregions (Ecoregion level 2) have higher vulnerability, i.e., are more consistently projected to experience changes in precipitation than has been observed since 1901 on a decadal basis. In contrast, major portions of Arizona, Washington, Oregon, and California have areas with low vulnerability to change with respect to precipitation. The PET vulnerability map is similar to the temperature vulnerability map, which is not surprising since the Hamon (1963) method of calculating monthly PET uses temperature as the major input. The 1 April snow accumulation (snow water equivalent) vulnerability map shows high vulnerability in many mountainous areas throughout the west. This seems to indicate that snow accumulation will change, particularly in transitional areas, compared to the most snow-prone areas of the west. *S′* is a measure of available water (excess water available for soil infiltration or overland flow) and has less spatial uniformity of vulnerability than temperature or PET. The map for *S′* suggests that the Warm Desert and Marine West Coast Forest Ecoregions are more likely to experience substantial changes in available water (i.e., high vulnerability) in the future. The FMI is calculated from the ratio of PET and precipitation as per Eq. (1). The FMI vulnerability map indicates that the Level-2 western Cordillera Ecoregion through northern Idaho (Fig. 1), a band of the western Cordillera running north and south through west of central Washington and Oregon (which includes portions of the Cascade Range), and portions of the cold desert ecoregions in southeastern Washington and north-western Arizona (Fig. 1) are more likely to see substantial changes to the FMI. The regional time series analyses (below) provide more information about whether those areas are expected to become wetter or drier. The seasonality vulnerability map identifies AUs that are likely to have changes in seasonality. Portions of the western Cordillera Ecoregion (Fig. 1, which includes the Sierra Nevada in California, the Cascade Mountains in Washington and Oregon, and transitional terrain in Idaho) are projected to be more vulnerable to changes in seasonality. Otherwise, large portions of the study area are not projected to be vulnerable to changes for seasonality.

#### 3.2.1 Vulnerability of hydrologic landscapes

Table 4 summarizes an analysis of the HL classifications of the most vulnerable AUs for each metric. For example, 75% of the AUs identified as vulnerable for snow accumulation (SWE) were classified as dry, moist, or wet, and therefore very wet, semi-arid, and arid AUs are less likely to be vulnerable to changes in snow accumulation. Likewise, 76% of AUs vulnerable to changes in seasonality had a spring seasonality during the 1971–2000 normal period. The physical properties represented by the dominant HL classes in Table 4 could help determine how various climate vulnerabilities are ultimately expressed. For example, vulnerability to changes in snow or FMI mostly occur in regions with wetter climates (moist, wet, or very wet climate), with fall or spring seasonality, in areas with low subsurface permeability. This could result in increased precipitation, with quicker runoff in areas that currently have delayed release of water. Similarly, areas vulnerable to changes in surface runoff are arid landscapes with winter seasonality and highly permeable subsurface parent materials. This means that these changes in runoff could have a large impact on subsurface recharge and, ultimately, baseflow.

#### 3.2.2 Case studies and locational time series

**Figure A2.**
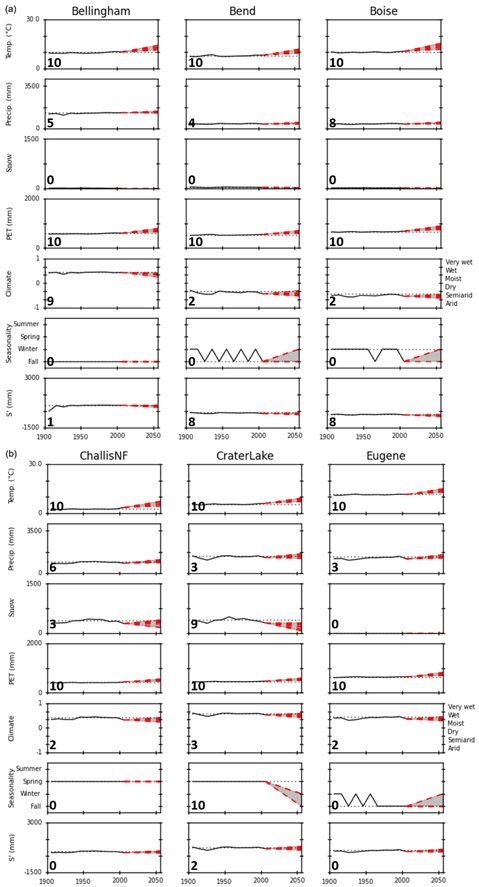

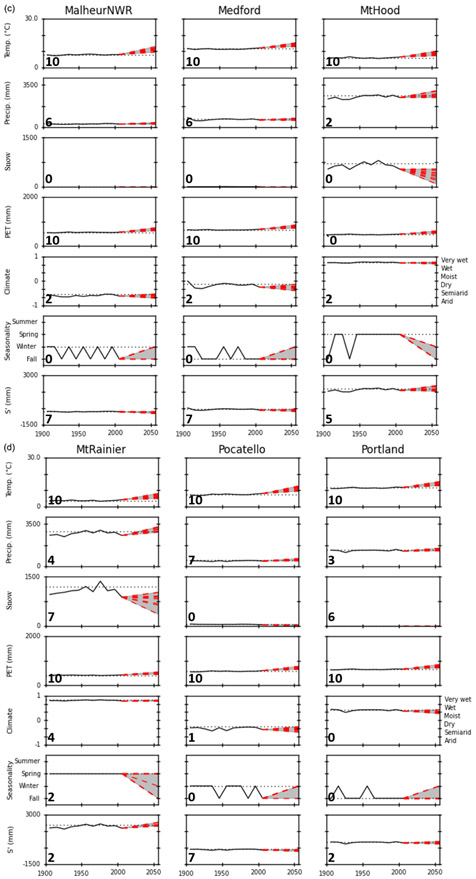

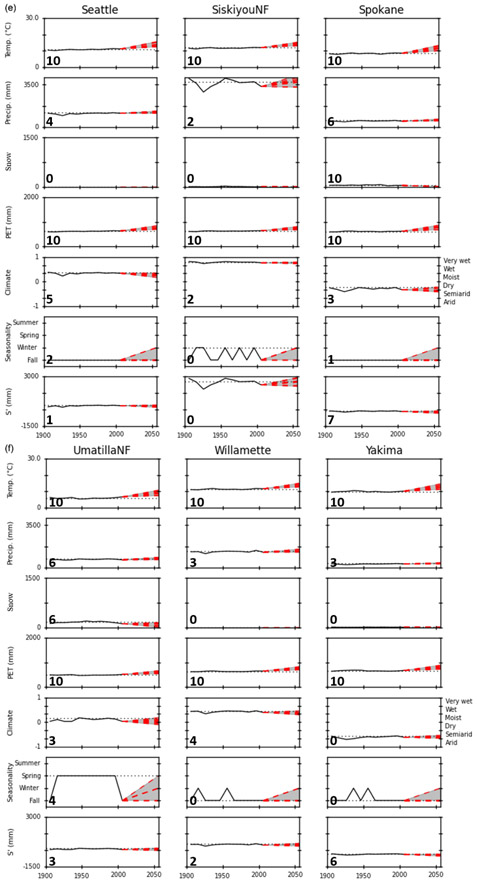

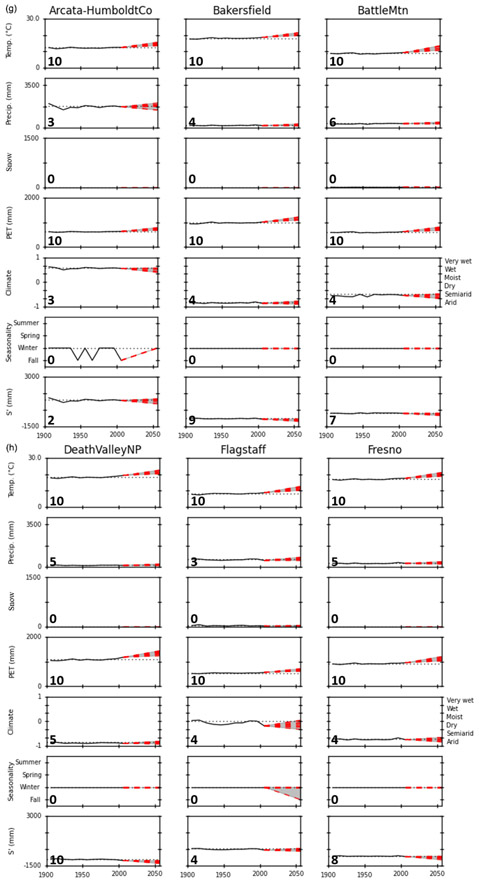

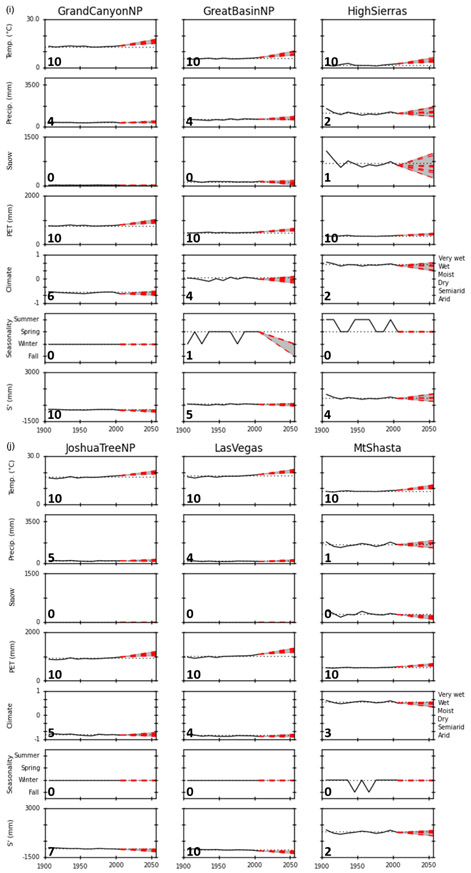

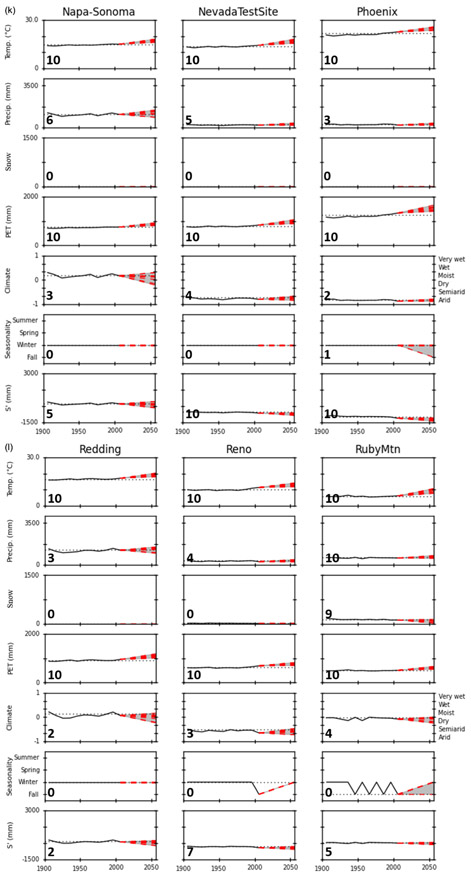

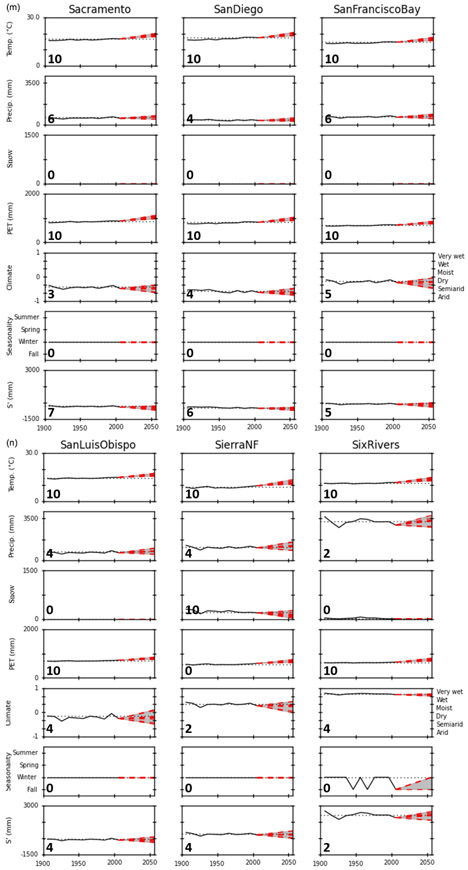

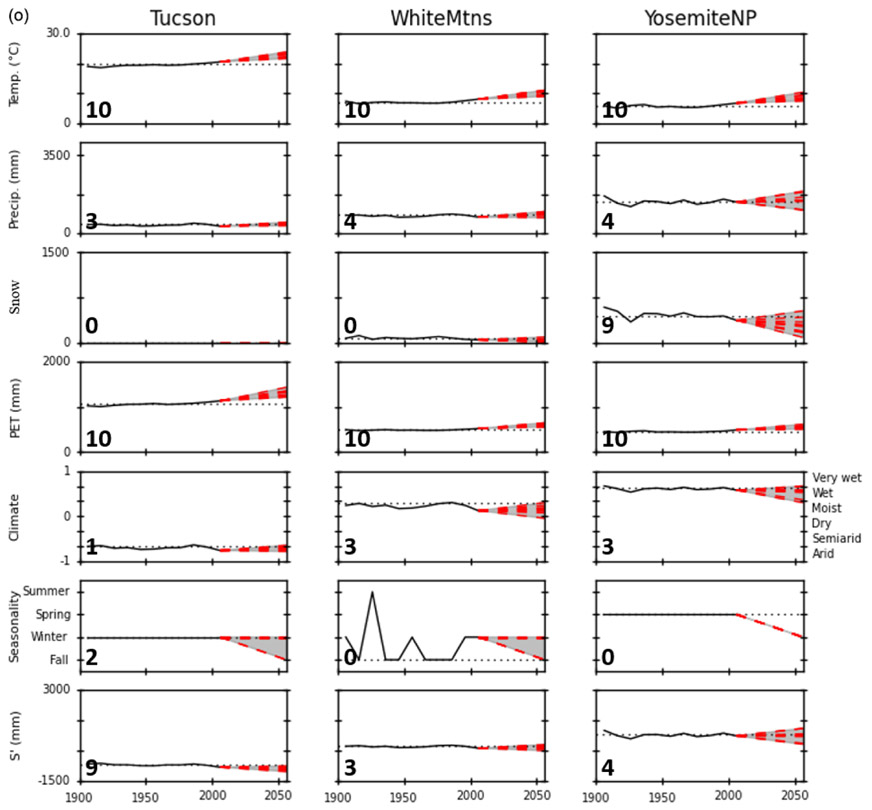
Time series of average decadal temperature, precipitation, snow (1 April snow water equivalent), potential evapotranspiration (PET), climate (FMI), seasonality, and available water (*S′*) for 45 specific locations in the western U.S. For the climate/FMI figures, the FMI values range from 1 to −1 (primary *y* axis on the left), whereas the categorical version of the index ranges from arid to very wet (secondary *y* axis on the right). Dotted black line represents the 1971–2000 base period; the dashed red line connects the 2001–2010 value to the 2041–2070 climate projections for each of the 10 models. The gray shaded area represents the range of model projections. The number in the lower left indicates the HLVA vulnerability index for the metric and location depicted in the associated graph. Note that Oregon, Washington, and Idaho locations are displayed first in alphabetical order and are followed by those of California, Nevada, and Arizona.

Hydrologic vulnerability analyses have been performed for a total of 45 exposure areas of ecological, economic, or social significance (Fig. 1 and Table 2; see Appendix A, Fig. A2). The vulnerability index for each location is also listed in Table 2 for each metric. Three case study locations that are of economic interest are explored in detail and include Mt. Hood (site no. 7), the Willamette Valley (site no. 9), and the Napa–Sonoma Valley (site no. 28). During the normal period, 61% of the 1867 km^2^ Napa–Sonoma Valley had an MwHMH HL classification, and thus much of the area was classified as having a moist climate with winter seasonality, high subsurface permeability, mountain terrain, and high surface permeability. Eighty-three percent of the 1234 km^2^ Willamette Valley AUs had an HL code of WfHTH during the normal period. Overall, the Willamette Valley had a wet climate dominated by fall seasonality, high subsurface permeability, transitional terrain, and high surface permeability. Table 2 indicates that 81% of the 834 km^2^ area analyzed for Mt. Hood had an HL code of VsHMH (very wet climate with spring seasonality, high subsurface permeability, mountainous terrain, and high surface permeability).

**Figure 5.**
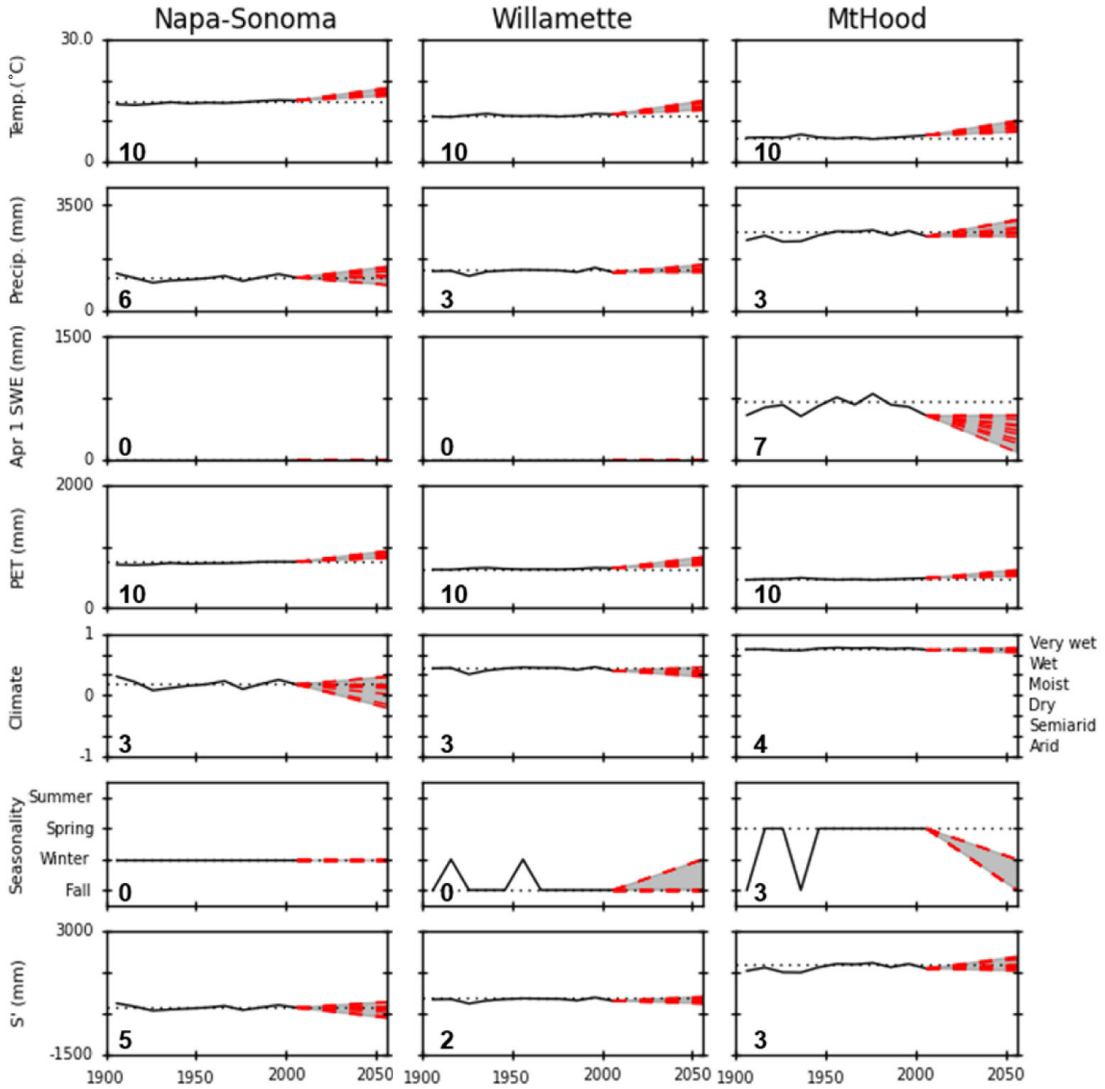
Time series of average decadal temperature, precipitation, snow (1 April snow water equivalent – mm), potential evapotranspiration (PET), climate (FMI), seasonality, and available water (*S′*) for three specific locations in the western U.S. For the climate/FMI figures, the FMI values range from 1 to −1 (primary *y* axis on the left), whereas the categorical version of the index ranges from arid to very wet (secondary *y* axis on the right). Dotted black line represents the 1971–2000 base period; the dashed red line connects the 2001–2010 value to the 2041–2070 climate projections for each of the 10 models. The gray shaded area represents the range of model projections. The number in the lower left indicates the vulnerability index for the metric and location depicted in the associated graph.

Figure 5 depicts line graphs of the historic and projected changes for the three case study locations (Mt. Hood, site no. 7, Willamette Valley, site no. 9, Napa–Sonoma Valley, site no. 28). The number in the lower left corner of each graph in Fig. 5 indicates the vulnerability index for the specific metric and location. For instance, precipitation at Mt. Hood has a vulnerability index of “3”, which indicates that three of the climate projections exceed the threshold of 2 standard deviations from the historic mean.

The time series in Fig. 5 (and Fig. A2) illustrate the trend in average decadal temperature, precipitation, SWE, PET, climate, seasonality of water surplus, and *S′*. Note that each future (2041–2070) climate projection is represented by a single data point that characterizes the 2041–2070 30-year range and is connected in Fig. 5 to the 2001–2010 decade with a dotted red line. Additional figures for 42 other locations are provided in Appendix A (Fig. A2). Given that Figs. 5 and A2 represent case study examples, Figs. 4 and S6 provide better insight into the spatial distributions of the vulnerability assessments for the western and continental U.S. Each of the three example case studies is predicted to be warmer in the 2041–2070 future climate projections. Further, these projected temperatures are almost always outside of the historic (1901–2010) temperature range, and so all locations have high vulnerability with respect to future temperatures. None of the three case studies shows a strong trend relating to future precipitation projections. Mt. Hood appears to exhibit increasing precipitation since 1901, but there is no evidence that the projected increases in precipitation are outside of historic behavior, and so the site has low vulnerability for that metric. Napa–Sonoma and the Willamette Valley have low vulnerability for change in snow, while Mt. Hood has high vulnerability for April 1 snow water equivalent in the 2041–2070 period. PET is calculated directly from temperature, and so its vulnerability is strongly correlated with temperature. There are no obvious trends in *S′* for the future projections in the three case studies; vulnerability of these sites for *S′* is low to moderate. The FMI projections for the Napa–Sonoma Valley, the Willamette Valley, and Mt. Hood are outside of 2 standard deviations of historical trends in 3 to 4 out of 10 of the projections (Table 2). In terms of seasonality, the vulnerability index is equal to zero in the Willamette and Napa–Sonoma valleys. For Mt. Hood, vulnerability is low, with all the future climate projections indicating that there will no longer be spring seasonality (the predominant historical season for runoff). Only three climate models suggest that decadal seasonality would transition to winter seasonality, which has not occurred since at least 1901.

## 4 Discussion

### 4.1 Analyses of retrospective and projected climate and hydrologic vulnerability

Vulnerability maps (Fig. 4) were developed to facilitate long-term planning for stakeholders for assessing their risk of climatic impacts. It is possible that ecosystems, businesses, and communities in areas mapped as vulnerable may struggle to adapt to stresses imposed by future environmental conditions. As mentioned previously, the vulnerability index offers no information about the directions of change projected by the 10 different models. Further, the RCP 8.5 pathway was selected because it most closely resembles observed conditions (Schwalm et al., 2020).

The consistently projected high temperature vulnerability could lead to problems related to heat stress (e.g., human-related physical and mental health issues), urban heat islands (particularly in areas with little tree cover), and other temperature-related problems (USGCRP, 2018). PET vulnerability would be problematic for agricultural systems, forest disease, and sectors that are drought sensitive (USGCRP, 2018). Precipitation vulnerability maps are important in specific areas with regards to flooding, landslides, and drought sensitivities. The vulnerability maps for snow accumulation and *S′* (surplus water available for runoff or infiltration) show that the areas mapped as most vulnerable for the two metrics are almost reversed, other than central Idaho and the coastal areas of California, Oregon, and Washington. According to the snow vulnerability map, it appears that most areas that receive large amounts of snow are projected to experience significant changes in future snow accumulation. In a related study on snow cover, Nolin and Daly (2006) found that the areas with the warmest winter temperatures are most at risk of having no snow cover in the future. Areas vulnerable for snow could impact not only the ski industry, but also water supply and streamflows, while the surplus water availability (*S*′) vulnerability metric relates more directly to streamflow and flooding. Most of the study area is not vulnerable to changes in FMI (Fig. 4), which is an assessment of overall water availability, although some areas are more vulnerable (the Willamette Valley in Oregon, east of Puget Sound in Washington, and the northern panhandle in Idaho). The vulnerability map for seasonality (Fig. 4) shows that portions of the western Cordillera (Fig. 1), including the high Sierra Nevada in California, the Cascade Mountains in Oregon and Washington, and the mountainous areas in Idaho, have higher vulnerability indices, which indicates susceptibility regarding water supply, flooding, and streamflows.

Our retrospective analysis of PRISM time series data provided an understanding of environmental conditions since 1901. We are aware of a few that have used retrospective analyses to inform their mapping efforts (Deviney et al., 2006; Kim et al., 2011; O’Brien et al., 2004) but are not aware of studies that have mapped resource vulnerability at a large scale using such data. Our definition of vulnerability is based on agreement of climate models leading to conditions that are outside of historic ranges. Our hypothesis is that systems experiencing future climate conditions outside of the historic range will not have the capacity to adapt to future conditions and therefore are vulnerable. The vulnerability issue is complicated by the fact that these vulnerability maps (Fig. 4) do not show how downstream areas could be impacted by these changes.

These vulnerability factors may be of interest to resource managers and decision makers, some of whom might consider high vulnerability for a single metric to be problematic. Yet for others, the additive or multiplicative impacts of numerous vulnerabilities may be of greater concern. For example, urban areas might be more impacted when vulnerable to multiple metrics, whereas PET vulnerability could be detrimental to agricultural or forested areas. Similarly, changes in seasonality from a snow-dominated system to rain could have profound implications across many sectors.

For this analysis, the 30-year normal climate conditions were compared to decadal climate conditions since 1901. In addition, the 30-year normals for future projections (2041–2070) were compared to the historic range of decadal climate data. While comparing 30-year normals in a decadal analysis might appear to be a discrepancy in the analysis, the intention was to conservatively quantify vulnerability indices. Thirty-year normals exhibit less variability than decadal averages or annual averages. By comparing decadal averages to the 30-year future climate normals, we are not treating past data the same as future climate projections. However, the resulting vulnerability conclusions are conservative, because if we had used decadal projections for future climate data, variability in the range of output would have increased and our vulnerability indices could have increased for all parameters.

### 4.2 Hydrologic response and hydrologic landscape classification

The HL class for an AU can provide insight into its hydrological response, given changes in the quantity (FMI) or timing of surplus water (seasonality) on a landscape. Yet these factors only account for a portion of the water balance. However, when moisture is available as surface runoff, it may infiltrate into the ground or act as surface runoff, depending on the HL surface permeability class. Water may enter and flow through the subsurface layers (depending on the HL subsurface permeability) towards a stream channel. If the water was directed as surface or subsurface runoff, it may be transported more quickly in the downhill direction and into a stream channel depending upon the HL terrain class, which governs steepness. As it relates to streamflow, the unique combination of the five HL characteristics (climate, seasonality, surface permeability, subsurface permeability, and terrain) allows for the hydrologic response to be assessed relative to changes in temperature and climate (Leibowitz et al., 2014; Patil et al., 2014). At its coarsest application as it relates to this study, the transition from spring to winter seasonality for the Mt. Hood case study would result in a shorter ski season with snow conditions that could be less ideal for winter sports. However, this transition would also have many downstream impacts that could include flooding or habitat impacts. The HL approach could also be used to determine any relationships between HL characteristics and hydrologic vulnerability, while case studies can show how the HLVA could be useful.

### 4.3 Case studies

Case studies are useful for illustrating how future climate conditions may impact important economic and conservation resources. It is necessary for a stakeholder to understand the parameters most important to their ecosystem, industry, or resource of interest, so that they can utilize location-specific information about their potential climatic impacts (Glick et al., 2011; Lawler et al., 2010). In Fig. 5, case study examples (Mt. Hood, site no. 7, Willamette Valley, site no. 9, Napa–Sonoma Valley, site no. 28) demonstrate how the HLVA can assist in understanding how climate can impact important local water resources.

The wine and ski industries are important stakeholders in the western U.S. that may experience impacts from hydrological changes. The Napa–Sonoma and Willamette valleys are known for their vineyards and associated wineries. Regarding their HL characteristics, they differ in their FMI class (Willamette is wet, whereas Napa–Sonoma is moist) and their seasonality (Willamette has a fall seasonality, while Napa–Sonoma has a winter seasonality). Due to the importance of the pinot noir varietals in the Willamette Valley (Olen and Skinkis, 2018) and their temperature sensitivity (Burakowski and Magnusson, 2012; Jones et al., 2010), local viticulturalists are likely more concerned with changes in temperature than FMI. The Napa–Sonoma region is recognized for a variety of grape cultivars (Elliott-Fisk, 1993) that are less sensitive to temperature fluctuations (Jones et al., 2010). Both the Willamette Valley and Napa–Sonoma have temperature vulnerability indices of 10 out of 10, and both have FMI vulnerability indices of 3 out of 10 (Fig. 5). These indices suggest that both locations are projected to have future temperatures that are different than historic temperatures. However, the Willamette Valley pinot noir grapes are more sensitive to temperature than in the Napa and Sonoma valleys. In addition, while both locations have the same FMI vulnerability indices, Fig. 5 illustrates that FMI projections for Napa–Sonoma are much more variable than for the Willamette Valley. Thus, there is more uncertainty in the modeled water availability for Napa–Sonoma. These results suggest that a vintner growing warm-temperature grapes in the Willamette Valley may have more confidence in their investments relative to a vintner in Napa–Sonoma, where there is more uncertainty regarding long-term water availability.

The skiing industry is economically important, and the impact between a high- and low-snowfall year for the State of Oregon is USD 38.1 million, while California is estimated to lose more than USD 75 million in low-snow years (Burakowski and Magnusson, 2012). Mt. Hood is known for its winter snow sports and tourism and would be impacted differently by the seven metrics than the Willamette and Napa–Sonoma case studies (Fig. 5). Thus, resource managers and business leaders at Mt. Hood are likely more concerned about snow accumulation in their watershed than those in the wine and grape industries (although a grape grower’s ability to irrigate may be impacted by snow accumulation in the region). According to our analyses, Mt. Hood is generally characterized by having a spring seasonality and has a snow vulnerability index of 7 out of a maximum of 10. Also, the analysis of HL seasonality suggests some chance of a shorter ski season due to the risk of spring runoff occurring earlier and imposing on the winter season. Even though these conditions have occurred in the past (Fig. 5), this may be much more deleterious to the economics of the future ski industry than it was in the 1900s, because it contributed much less to the historic economy (for additional examples, refer to Appendix A2).

## 5 Summary and conclusions

The hydrologic landscape (HL) concept is useful for gaining a better understanding of hydrologic behavior at the assessment unit and watershed scales across large geographic regions. By applying the HL concept to climatic and vulnerability analyses, we provide a planning approach that allows resource managers to determine how vulnerable their location is to changes associated with climate that are important for a particular industry or application. Assessment of expected hydrologic response based upon physical and climatic characteristics has the potential to offer further insight into the idiosyncrasies of the threats faced by a stakeholder or industry across large geographic areas. This will allow them to make informed decisions about the risk imposed by potential changes that could affect their long-term planning efforts. The methodology also allows stakeholders to focus on specific areas of interest, which provides the flexibility necessary for the information to be relevant across applications and sectors. Examples of another phenomenon that could be examined using a similar or modified approach could include vulnerability associated with wildfire, landslides, snowmelt-related flooding, wetland persistence, and flow permanence, among others. Other industries that could also be analyzed could include those associated with water-reliant industries, such as agriculture (timber, fruit crops, seed crops, etc.), freshwater fisheries, and winter-tourism industries. By applying the modified Wigington et al. (2013) approach across the western U.S., resource managers will be able to base management decisions on assessments of climatic impacts of water resource-related vulnerabilities.

## Supplementary Material

Sup1

## Figures and Tables

**Table 1.** CMIP5 Climate Model summary for 2041–2070 precipitation and temperature data (Bureau of Reclamation, 2014).

WCRP CMIP5 Climate Model	Model abbreviated name	Model realization used herein	Abbreviated name used in Fig. 3 for realization
Canadian Earth System Model	CanESM2	r5i1p1	CanESM2
Community Climate System Model	CCSM4	r1i1p1	CCSM4
Community Climate System Model	CCSM4	r4i1p1	CCSM4-R4
Community Earth System Model	CESM1	r3i1p1	CESM1
Commonwealth Scientific and Industrial Research Organisation Mark 3.6	CSIRO-Mk3-6-0	r5i1p1	CSIRO
Geophysical Fluid Dynamics Laboratory Coupled Climate Model	GFDL-CM3	r1i1p1	GFDL
Hadley Global Environment Model	HadGEM2-AO	r1i1p1	HadGem
Institute for Numerical Mathematics Climate Model	INM-CM4	r1i1p1	inmcm4
Model for Interdisciplinary Research on Climate	MIROC-ESM	r1i1p1	MIROC
Meteorological Research Institute	MRI-CGCM3	r1i1p1	MRI-CGCM3

**Table 2.** Summary table for 45 study locations (sorted by decreasing latitude) providing a numeric ID from Fig. 1, total analysis area, dominant HL class (representing climate, seasonality, subsurface permeability, terrain, and surface permeability), percent area represented by the dominant HL class, latitude and longitude of the center point of the area, and vulnerability indices for temperature, precipitation, potential evapotranspiration (PET), surplus water (*S′*), snow water equivalent (snow), Feddema Moisture Index (FMI), and seasonality.

Site no.	Name	Area (km^2^)	Dominant HL class*	Dominant % area	Coordinates		Vulnerability index						
					Lat.	Long.	Temp.	Precip.	PET	*S′*	Snow	FMI	Seasonality
1	Bellingham	212	WfLTH	99%	48.77	−122.45	10	5	10	1	0	9	0
2	Spokane	592	DfHTH	80%	47.64	−117.43	10	6	10	7	10	3	1
3	Seattle	669	WfLTH	78%	47.60	−122.25	10	4	10	1	0	5	2
4	Mt. Rainier	718	VsLMH	76%	46.85	−121.79	10	4	10	2	7	4	2
5	Yakima	438	SfHTH	86%	46.63	−120.60	10	3	10	6	0	0	0
6	Portland	932	WfHTH	67%	45.53	−122.66	10	3	10	2	0	6	0
7	Mt. Hood	834	VsHMH	81%	45.37	−121.70	10	3	10	3	7	4	3
8	Umatilla NF	2147	MsLMH	29%	44.87	−118.70	10	6	10	3	6	3	4
9	Willamette	1234	WfHTH	83%	44.84	−123.14	10	3	10	2	0	4	0
10	Challis NF	4348	WsLMH	74%	44.55	−114.75	10	6	10	0	3	2	0
11	Bend	948	SfHTH	68%	44.21	−121.26	10	4	10	8	0	3	0
12	Eugene	523	WfHFH	64%	44.10	−123.15	10	3	10	1	0	2	0
13	Boise	594	SwHTH	51%	43.61	−116.24	10	8	10	8	0	2	0
14	Malheur NWR	1355	SwHFH	69%	43.27	−119.04	10	6	10	7	0	2	0
15	Crater Lake	1721	WsHTH	45%	42.98	−122.08	10	3	10	2	9	3	10
16	Pocatello	349	DwHTH	45%	42.88	−112.43	10	7	10	7	0	1	0
17	Siskiyou NF	926	VwLMH	100%	42.36	−124.29	10	2	10	0	0	2	0
18	Medford	375	DfLTH	60%	42.34	−122.89	10	1	10	5	0	2	0
19	Six Rivers	1527	VwLMH	100%	41.63	−123.79	10	2	10	2	0	4	0
20	Mt. Shasta	956	WwHMH	49%	41.36	−122.23	10	1	10	2	0	3	0
21	Ruby Mtn	1132	DfLTH	44%	40.68	−115.31	10	6	10	5	9	4	0
22	Arcata-Humboldt Co	2511	WwLMH	63%	40.62	−124.01	10	3	10	2	0	3	0
23	Redding	478	MwHTH	59%	40.56	−122.38	10	2	10	2	0	2	0
24	Battle Mtn	902	SwLMH	75%	40.09	−116.71	10	6	10	7	0	4	0
25	Reno	382	SwHTH	40%	39.54	−119.80	10	4	10	7	0	3	0
26	Great Basin NP	38	MsLMH	100%	39.01	−114.26	10	4	10	5	0	4	1
27	Sacramento	855	SwHFH	88%	38.57	−121.39	10	6	10	7	0	3	0
28	Napa–Sonoma	1867	MwHTH	61%	38.37	−122.53	10	6	10	5	0	3	0
29	Yosemite NP	2455	VsLMH	44%	37.93	−119.55	10	4	10	4	9	3	0
30	San Francisco Bay	3356	DwHMH	19%	37.44	−122.29	10	6	10	5	0	5	0
31	Sierra NF	5349	WwLMH	31%	37.17	−119.05	10	4	10	4	0	2	0
32	High Sierras	2239	WsLMH	32%	37.15	−118.81	10	2	10	4	1	2	0
33	Nevada Test Site	3121	AwHMH	67%	36.96	−116.22	10	5	10	10	0	4	0
34	Fresno	1393	AwHFH	100%	36.74	−119.91	10	5	10	8	0	4	0
35	Death Valley NP	7862	AwHMH	50%	36.45	−117.03	10	5	10	10	0	5	0
36	Las Vegas	977	AwHTH	65%	36.23	−115.26	10	4	10	10	0	4	0
37	Grand Canyon NP	3475	SwHMH	28%	36.22	−112.11	10	4	10	10	0	6	0
38	San Luis Obispo	2653	DwLMH	98%	35.36	−120.63	10	4	10	4	0	4	0
39	Bakersfield	3399	AwHFH	96%	35.33	−119.14	10	4	10	9	0	4	0
40	Flagstaff	365	DwHMH	51%	35.19	−111.60	10	3	10	4	0	4	0
41	Joshua Tree NP	2599	AwLMH	68%	33.92	−115.99	10	5	10	7	0	5	0
42	White Mtns	4855	WfLMH	23%	33.87	−109.53	10	4	10	3	0	3	0
43	Phoenix	2304	AwHFH	63%	33.52	−112.11	10	3	10	10	0	2	1
44	San Diego	1276	SwLMH	37%	32.90	−117.06	10	4	10	6	0	4	0
45	Tucson	1838	AwHTH	62%	32.19	−110.95	10	3	10	9	0	1	2

* Climate class (1st letter): V: very wet; W: wet; M: moist; D: dry; S: semi-arid; A: arid. Seasonality class (2nd letter): f: fall; w: winter; s: spring; u: summer. Subsurface permeability class (3rd letter): L: low; H: high. Terrain class (4th letter): M: mountain; T: transitional; F: flat. Surface permeability class (5th letter): L: low; H: high.

**Table 3.** Percent of area of each HL category and classification within the six-state region (1971–2000).

Category	Classification	Area (%)
Climate	Arid	21%
	Semi-arid	34%
	Dry	15%
	Moist	9%
	Wet	14%
	Very wet	7%
Season	Spring (AMJ^1^)	13%
	Summer (JAS^2^)	1%
	Fall (OND^3^)	24%
	Winter (JFM^4^)	63%
Subsurface permeability	Low	40%
	High	60%
Terrain	Flat	7%
	Transitional	63%
	Mountain	30%
Surface permeability	Low	2%
	High	98%

1 AMJ: April, May, and June.

2 JAS: July, August, and September.

3 OND: October, November, and December.

4 JFM: January, February, and March.

**Table 4.** Hydrologic landscape characteristics of assessment units identified as vulnerable (having a vulnerability index greater than 7 on a scale of 10) for each metric.

		% assessment units that share HL classification									
		Climate^1^		Seasonality^2^		Subsurface permeability^3^		Terrain^4^		Surface permeability^3^	
Vulnerability parameter	Temperature	70%	D, S, or A	87%	f or w	60%	H	93%	M or T	98%	H
	Precipitation	72%	D or S	79%	f or w	71%	H	97%	M or T	98%	H
	PET	70%	D, S, or A	87%	f or w	60%	H	93%	M or T	98%	H
	Surplus water (*S′*)	92%	A or S	79%	w	75%	H	87%	M or T	99%	H
	Snow water equivalent (SWE)	75%	D, M, or W	87%	f or s	53%	L	82%	M	100%	H
	FMI	71%	V or W	65%	f	75%	L	75%	M	100%	H
	Seasonality	75%	W or M	76%	s	51%	H	83%	M	99%	H

1 A: arid, S: semi-arid, D: dry, M: moist, W: wet.

2 f: fall, w: winter, s: spring.

3 L: low, H: high.

4 T: transitional, M: mountainous.

## Data Availability

The geospatial data files that could be used to recreate the published products associated with this paper are available via the U.S. Environmental Protection Agency’s Environmental Data Gateway (Jones and Leibowitz, 2021; https://doi.org/10.23719/1522399).
