# Supplementary material for: Using hydrologic landscape classification and climatic time series to assess hydrologic vulnerability of the western U.S. to climate
Source: Hydrol Earth Syst Sci. Author manuscript; Available in PMC 2022 Jun 11. (PMC8353951; doi:10.5194/hess-25-3179-2021)
Supplement: Sup1 [file NIHMS1714591-supplement-Sup1.docx]

*Supplement of*

Using hydrologic landscape classification and climatic time series to assess hydrologic vulnerability of the Western U.S. to climate

Chas E. Jones Jr. et al.

*Correspondence to:* Chas E. Jones Jr. ([chas@chasjones.com](mailto:chas@chasjones.com))


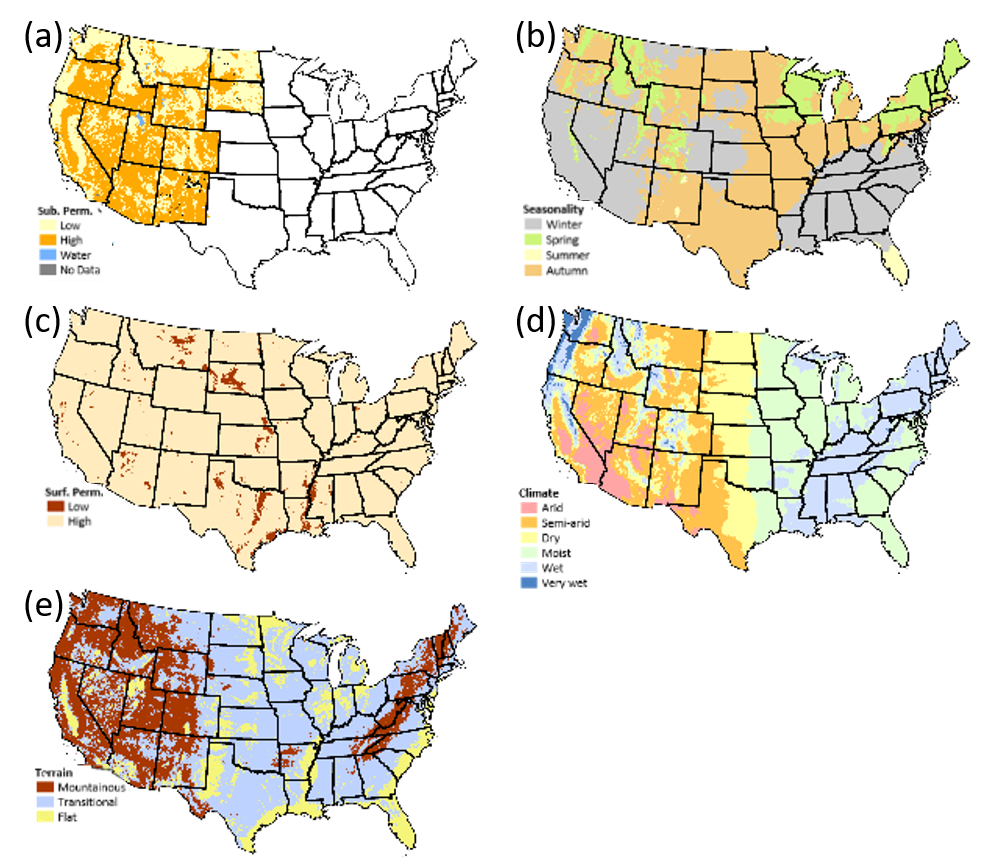
Figure S1. Hydrologic Landscape maps of the United States that were used in the HLVA analysis [(a) Subsurface Permeability, (b) Seasonality of precipitation surplus, (c). Surface permeability, (d) Climate, and (e) Terrain]. Notes: The seasonality map for the PNW has been updated from the original Leibowitz 2016 HL map, as we separated their winter seasonality into two seasons (winter and fall). In addition, the subsurface permeability maps were only completed for the western most portions of the U.S.


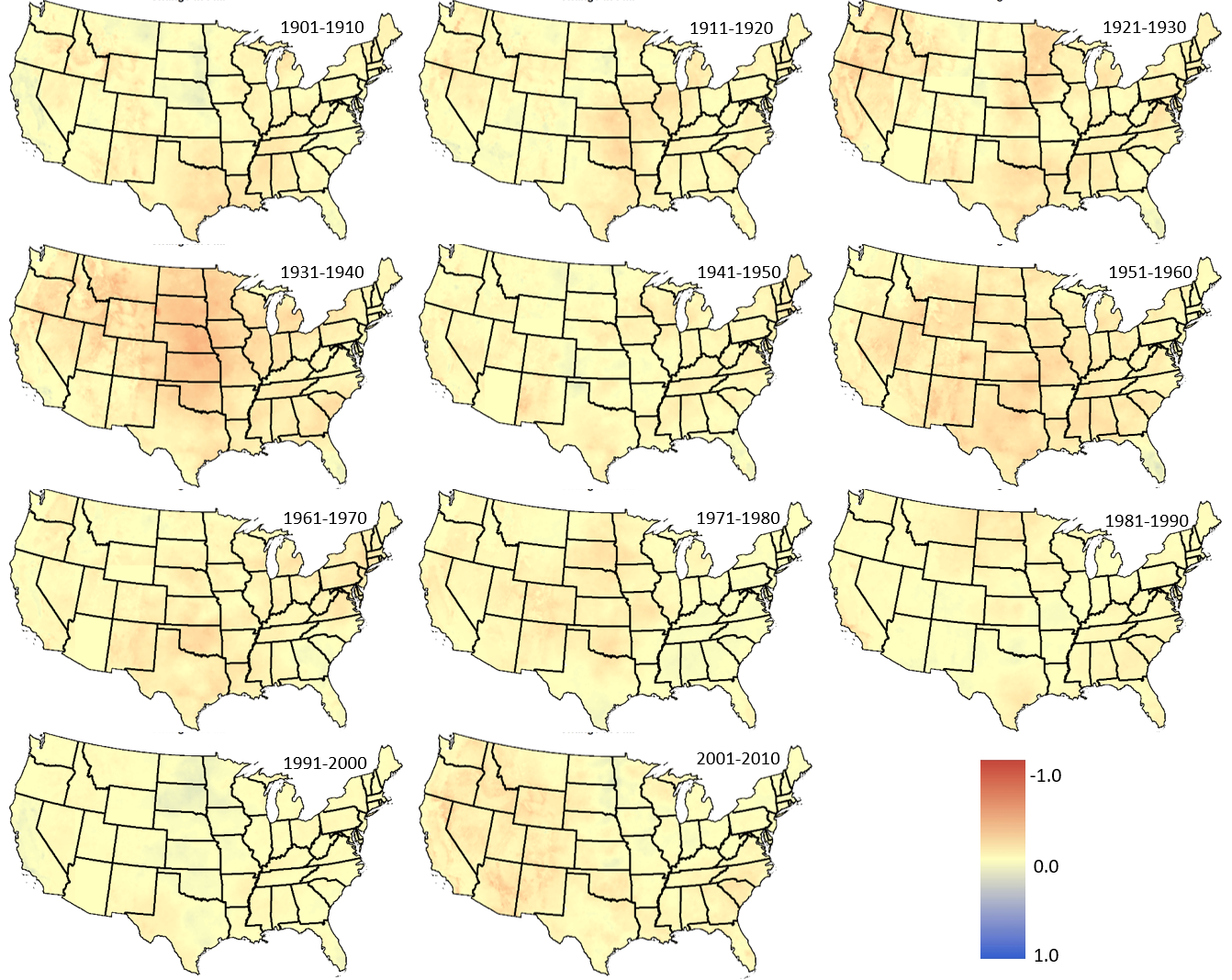
 Figure S2. Decadal change in Feddema Moisture Index relative to 1971–2000 normal period. Red and blue colors indicate drier and wetter average conditions than 1971–2000, respectively.


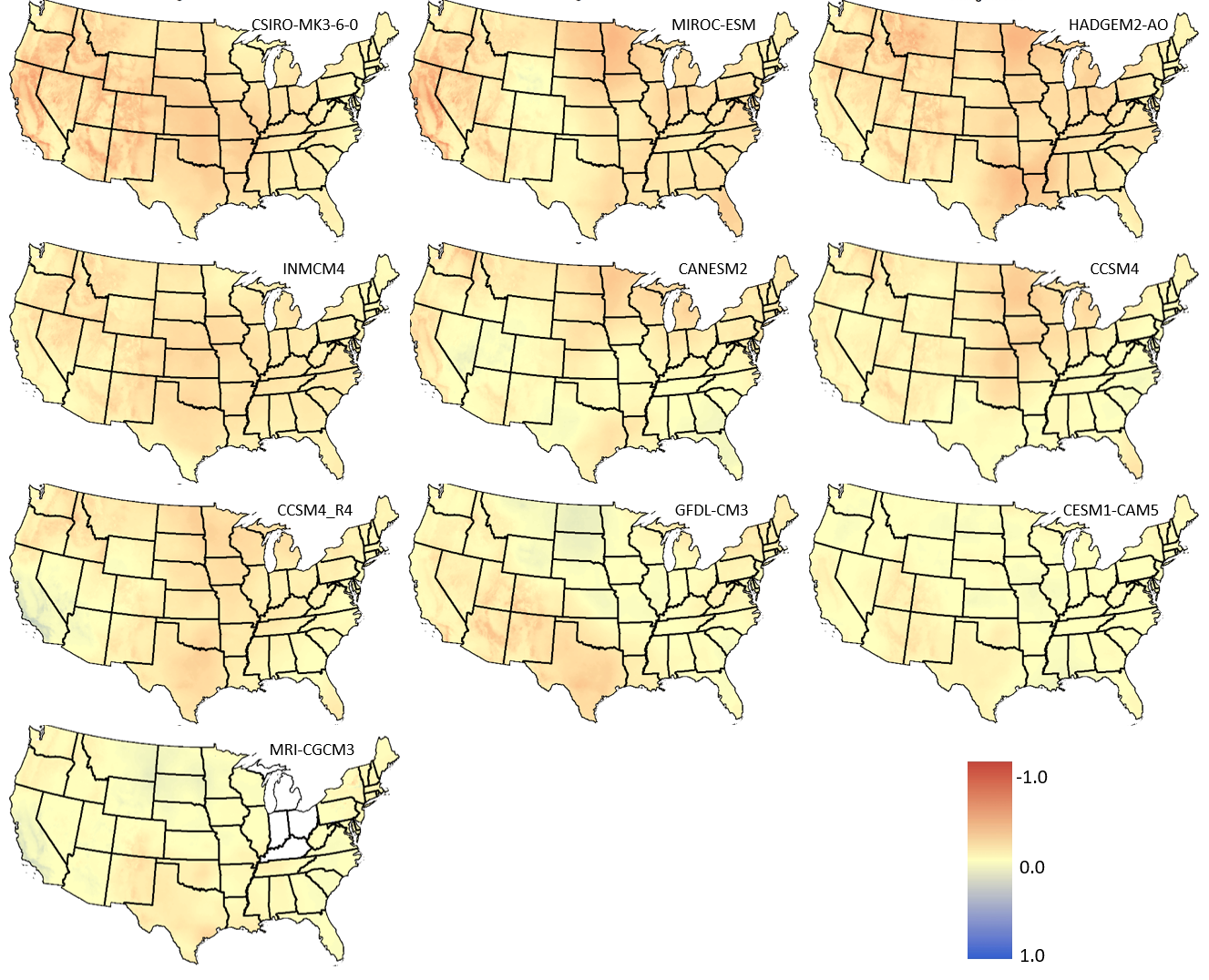


Figure S3. Projected change in Feddema Moisture Index for 2041–2070 relative to 1971–2000 for ten climate models. Red and blue colors indicate drier and wetter conditions than the 1971–2000 base period, respectively. Abbreviated model names correlate to those in Table 1.


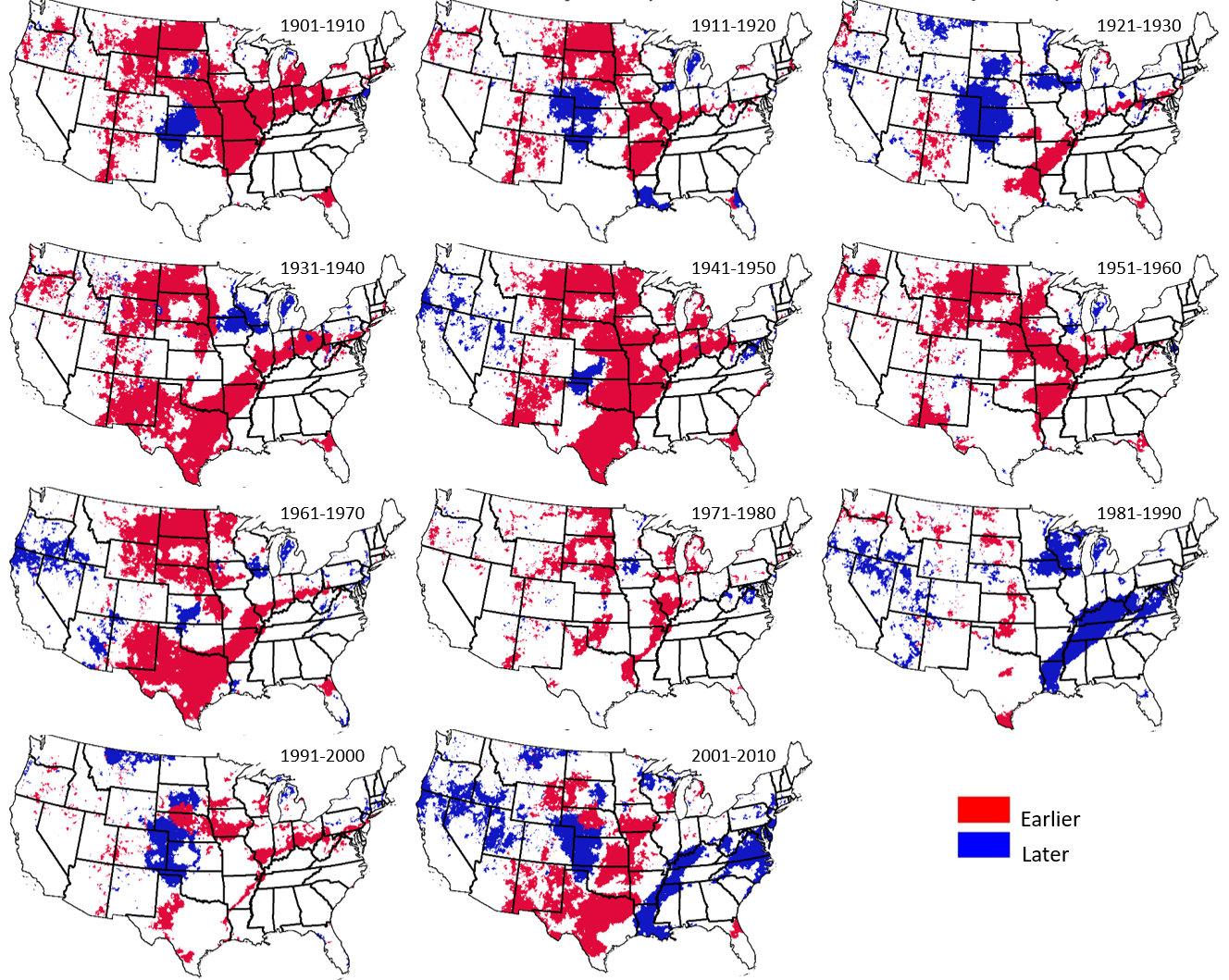
 Figure S4. Decadal change in seasonality of water surplus since 1901 relative to 1971–2000. Red and blue colors indicate earlier and later seasonality than the 1971–2000 base period, respectively.


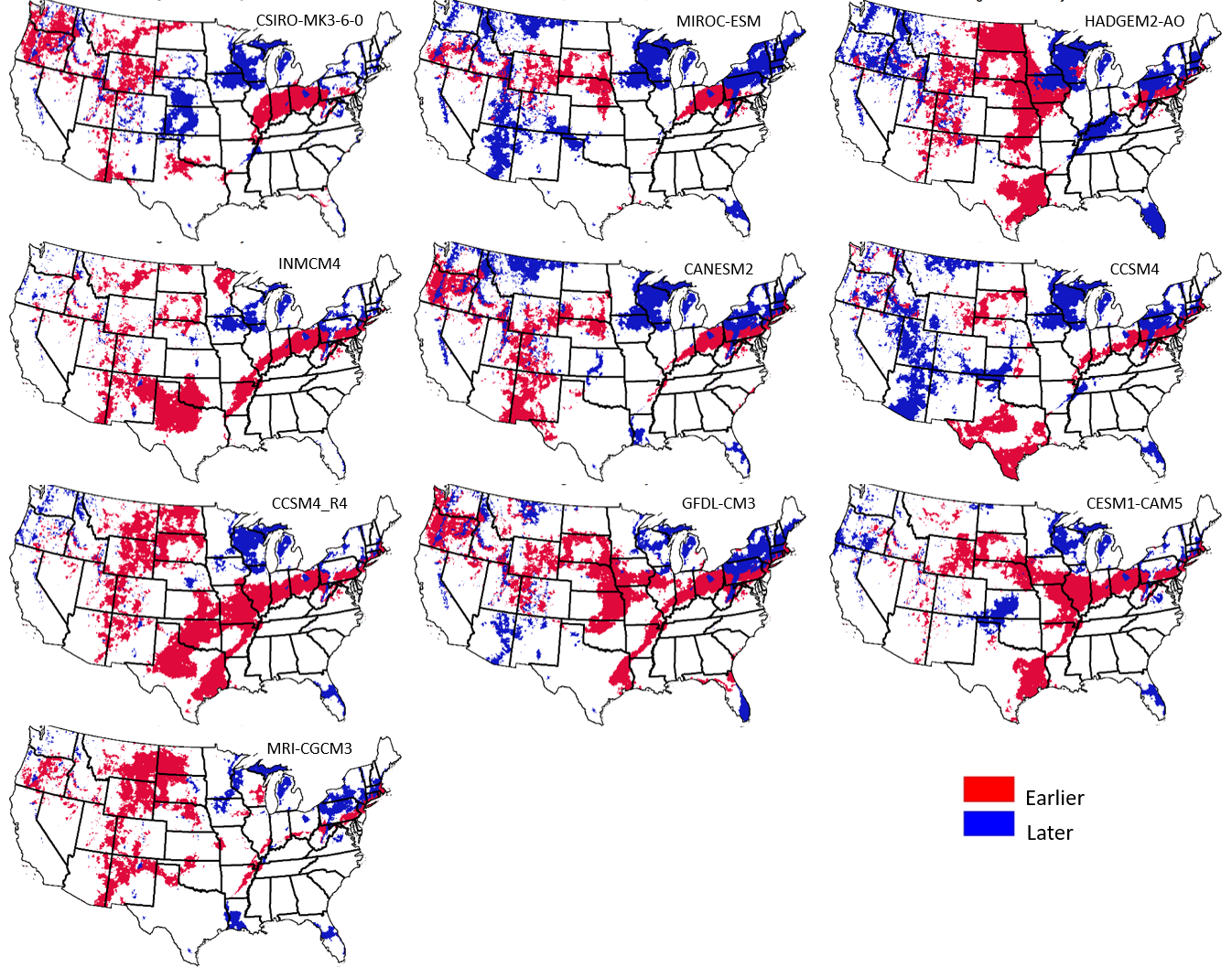
 Figure S5. Projected change in seasonality of water surplus for 2041–2070 relative to 1971–2000 for ten climate models. Red and blue colors indicate earlier and later seasonality than the 1971–2000 base period, respectively. Abbreviated model names correlate to those in Table 1.


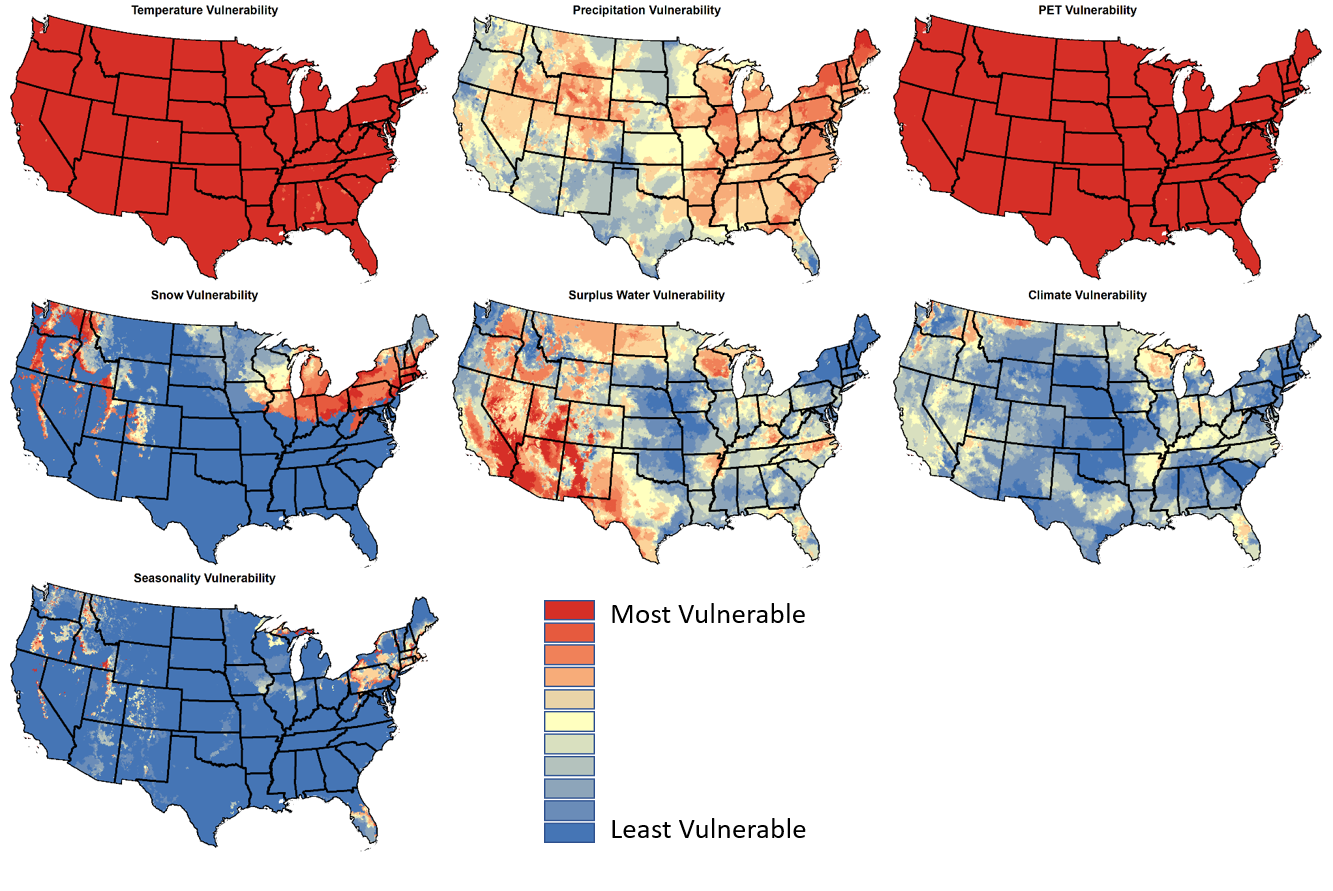


Figure S6. Vulnerability indices for temperature, precipitation, potential evapotranspiration, snow water equivalent (April 1), S’ (available water), Feddema Moisture Index, and seasonality. The least vulnerable locations are those projected to be within two-standard deviations of the historic (1901–201) mean in all nine climate models.
